# The Mott–Jones Electron Crystal: Patterning Atomic Positions for Pseudogap Formation in Hume–Rothery Phases

**DOI:** 10.1021/acs.inorgchem.6c02050

**Published:** 2026-07-06

**Authors:** Leah C. Garman, Daniel C. Fredrickson

**Affiliations:** Department of Chemistry, 5228 University of Wisconsin-Madison, 1101 University Avenue, Madison, Wisconsin 53706, United States

## Abstract

The valence electrons of Hume–Rothery intermetallics are envisioned to be so delocalized that together they approximate a free electron gas. However, electron count plays a key role in their structural preferences. This influence is generally attributed to the Mott–Jones (MJ) effect, in which the mixing of otherwise free electron states is induced by the presence of a periodic array of ions. Less clear, however, is how these interactions translate into the often complex local atomic configurations encountered in these materials. Here, we investigate this connection through the visualization of the partial electron densities associated with MJ planewave interactions. Over a series of structures with varying complexities, a simple mechanism emerges. In each case, essentially the same partial electron density distribution is obtained, which we refer to as the BCC-derived Mott-Jones electron crystal. The atomic centers are placed within channels in this density, corresponding to nodal surfaces in the underlying standing waves with different structures representing distinct ways of spreading atoms over the channels. These positions reinforce the splitting of *s*- and *p*-orbital character across pseudogaps, while the contributions of additional reciprocal lattice vectors fine-tune this effect. We comment on the potential existence of Mott–Jones electron crystals with other topologies.

## Graphical abstract

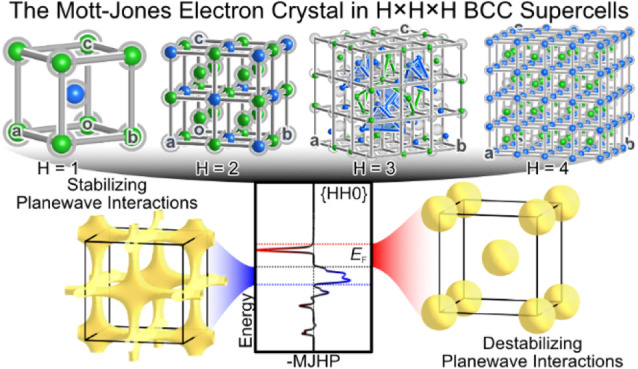

## 1 Introduction

The correlation of molecular shapes with electron count is one of the foundations of chemical science. For example, the geometries of deltahedron-based borohydrides,^1^ the carbon backbones of organic chemistry,^2^ hypervalent fluorides,^3^ and organometallic compounds^4^ can all be traced to the favorability of closed-shell electron configurations for specific atoms or clusters of atoms. In metals, however, preferred electron counts can arise not only from local atomic configurations but also from a very different aspect of a structure: lattice periodicity. This connection is explained by the Mott–Jones (MJ) model, which connects the wavelengths of the highest-energy electrons in a free electron gas to optimal periodicities for the atomic centers.^5–9^ An intriguing and essentially unexplained aspect of these compounds, though, is that intricate clustering of atoms can emerge from this seemingly simple resonance condition. In this article, we will trace the origins of these arrangements, illustrating how they are templated by an electron lattice formed from the frontier orbitals of these systems.

The MJ model, our starting point, is remarkable in how it relates preferred electron concentrations (the valence electron count per atom) to experimental observables, particularly diffraction data. Here, the intensity of a diffraction peak is qualitatively connected to the reciprocal lattice vectors for which the presence of atoms on the corresponding lattice planes will induce free-electron planewaves with opposite wavevectors (**k** and **–k**) to mix, leading to standing density waves whose wavecrests lie on or between atoms with an accompanying energy splitting. In this way, the most intense diffraction peaks can be used to predict the diameter of the Fermi sphere (Figure 1a) for the free-electron gas (a simple function of the number of valence electrons per atom), for which a large splitting between filled and empty electronic levels will open when interactions with the atomic centers are turned on.

**Figure 1:**
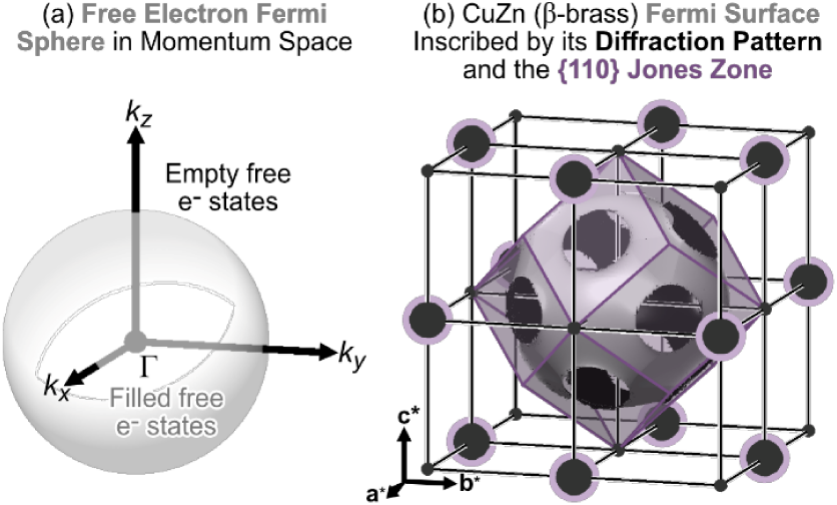
(a) The free-electron Fermi sphere. (b) The DFT-calculated Fermi surface for CuZn (β-brass) unwrapped in reciprocal space by the dominant planewaves, inscribed within the calculated diffraction pattern for CuZn with the most intense {110} peaks outlined in purple. The Jones Zone, defined by planes perpendicularly bisecting the {110} vectors, is drawn as a purple polyhedron. Adapted with permission from Garman, L. C.; Fredrickson, D. C. *J. Phys. Chem. C* **2024**, 128, 14442–14457. Copyright 2024 by the American Chemical Society.

This is illustrated in Figure 1b with the Fermi surface of β-brass, CuZn, unfolded so that each point of the surface is mapped to the position in reciprocal space of the wavefunction’s most dominant planewave. The surface is still largely spherical. However, there are circular holes where the surface bisects the strongly diffracting {110} family of diffraction vectors, outlined in purple. We emphasize this connection by inscribing the Fermi surface within the Jones Zone, a reciprocal space polyhedron constructed by perpendicularly bisecting the intense {110} vectors. The free-electron orbitals on the surface of this polyhedron are induced by this family of reciprocal lattice vectors to interact with their counterparts on the opposite faces.

While this model is extremely elegant, one might wonder how well it works in practice. In fact, it has been successfully used in explaining a wide range of compounds, including Hume–Rothery phases,^9–24^ quasicrystal approximants,^16,25–45^ and polar intermetallics.^46,47^ Even so, the evidence for the role played by this mechanism has been largely circumstantial, generally in the observation of an electronic pseudogap at the expected electron concentration.^17,48^

The observation of such pseudogaps does not directly verify the MJ effect or quantify its energetic role. For this reason, we recently developed the Mott–Jones Hamilton Population (MJHP) as a theoretical framework to analyze the contributions of the MJ effect to the overall energy and electronic structure of a system.^49^ The MJHP extracts the Hamilton populations for specific pairs of planewaves in the electronic structure, allowing their couplings from MJ effects to be easily detected and quantitatively assessed.

In applying this method to a series of compounds previously interpreted in terms of the MJ picture, we noted a simple, unifying trend (Figure 2). The compounds β-brass (CuZn), BiF_3_-type AlCu_3_, CaF_2_-type AuAl_2_, Ga_4_Ni_3_-type Al_4_Ni_3_, and γ-brass (Cu_5_Zn_8_) each exhibit strong MJ interactions associated with the {*HH*0} family of reciprocal lattice vectors, where *H* is an integer specific to each compound. In fact, as illustrated in Figure 2b, the *H* value points to the derivation of the compound from an *H* × *H* × *H* supercell of a body-centered cubic (BCC) parent structure, suggesting that the MJ effects in all cases are connected to this common subcell.

**Figure 2:**
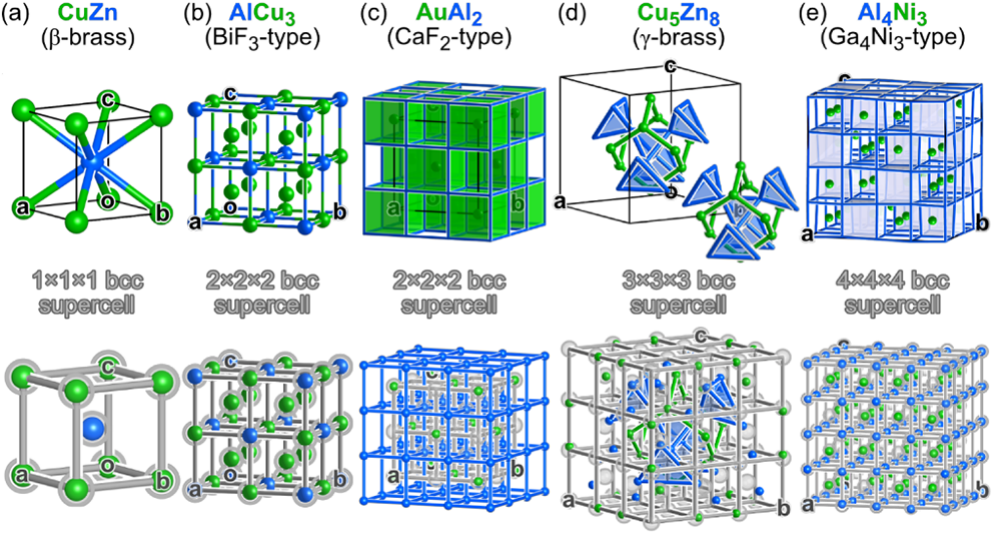
Gallery of superstructures of the body-centered cubic (BCC) arrangements explored in this work: (a) β-brass, CuZn, as a 1 × 1 × 1 BCC supercell. (b) BiF_3_-type AlCu_3_ and (c) CaF_2_-type AuAl_2_ as 2 × 2 × 2 BCC supercells. (d) γ-brass, Cu_5_Zn_8_, as a 3 × 3 × 3 BCC supercell, and (e) Ga_4_Ni_3_-type Al_4_Ni_3_ as a 4 × 4 × 4 BCC supercell. The top panel illustrates the crystal structure of these compounds, while the bottom panel highlights their connection to the BCC structure.

Herein, we pursue this clue to the structural mechanisms underlying the MJ effect. We will see that the planewave mixing induced by the reciprocal lattice vectors leads to the formation of periodic electron density distributions, qualitatively similar to charge-density waves,^50,51^ which is preserved in all of these structures. A key feature of this electron density, which we term the BCC-based Mott–Jones electron crystal, is the presence of channels created by the nodal surfaces of the occupied frontier orbitals that dictate potential placements of atoms within the crystal structure that optimize their *p*-orbital contributions to the bonding. This scheme accounts not only for the simple superstructures of the BCC arrangement but also for the cluster arrangements in the γ-brass structure, the prototype of complexity emerging from the MJ effect, through the simple sliding of atoms along these channels. Finally, we will generalize these results with the proposal of the existence of other Mott–Jones electron crystals derived from other families of reciprocal lattice vectors, each with their own series of potential crystal structures.

## 2 Experimental Section

### 2.1 Electronic Structure Calculations

The nonspin-polarized ground-state electronic structures of CuZn,^52^ AlCu_3_,^53^ AuAl_2_,^54^ Cu_5_Zn_8_,^55,56^ and Al_4_Ni_3_ ^57^ were calculated with the ABINIT^58–60^ software package using density functional theory (DFT) with the Perdew–Burke–Ernzerhof (PBE)^61^ parametrization of the generalized gradient approximation (GGA) and projector augmented wave (PAW)^62–64^ potentials. The standard Jollet–Torrent–Holzwarth (JTH) atomic data set was used for Cu,^65^ while data sets optimized for MJHP analysis (available with the *pawMJHP_analysis* package)^66^ were used for Al, Ni, Zn, and Au.

The crystal structures were geometrically optimized beginning with the relaxation of the atomic positions with fixed cell parameters and then proceeding to the release of both the atomic positions and the cell parameters. At each step of the algorithm, the self-consistent field (SCF) cycle was iterated (electronic step) until the convergence of the residuals on the potential between two sequential steps differed by less than 1.0 × 10^–10^. The geometry optimizations employed *k*-point meshes that converge the total energy to differences of <3 meV/atom. In preparation for the MJHP analysis, static calculations were performed on the optimized geometries in their conventional unit cells on a *k*-point mesh at least two times as dense as the original mesh to ensure a high resolution of the subtle effects near the Fermi energy (*E*_F_). The DOS distribution was also calculated on these dense *k*-point meshes using the tetrahedron method.^67^ Further details regarding the computational parameters, including *k*-point meshes, energy cutoffs, and optimized geometries, are given in the Supporting Information.

### 2.2 The PAW-MJHP Analysis

The role of the MJ effects in each structure was investigated with the MJHP analysis^49,66^ using the program *pawMJHP_analysis* ^68^ in several modes. For the MJHP­(*E*,2θ) analysis, the ground-state density from the single-point energy calculations was read for the simulation of the powder X-ray diffraction pattern. The reciprocal lattice vectors with appreciable diffraction intensity were then folded back into the Brillouin zone, and a nonself-consistent calculation was performed with ABINIT on the resulting high-symmetry *k*-points using the SCF ground-state density as input. The local effective potential, pseudo-wavefunctions, and atomic data sets were then processed for the analysis. The MJHP_HKL_(E) analysis and MJ electron density construction (described in Section 3.1), meanwhile, draw on the output of the SCF ground-state calculations.

The band structures and fat-band representations of the orbital character of the bands were generated from nonself-consistent calculations using the SCF ground-state density as input. The calculations were performed for a set of *k*-points along paths connecting the high-symmetry positions of the Brillouin zone. The results were plotted using a custom MATLAB script that is available as part of the *pawMJHP*_analysis package.

## 3 Results and Discussion

### 3.1 The Mott–Jones Electron Density

Previously, we introduced the Mott–Jones Hamilton population (MJHP) as a tool to identify and quantitatively assess the MJ effects in the opening of electronic pseudogaps in intermetallic systems. While this approach allowed us to detect energetic signatures for the MJ effects across a range of systems, as yet, the method does not reveal how these interactions are supported by atomic arrangements within the compounds. Here, we will see how this connection can be traced by extracting the portion of the electron density that is tied to the MJ effect.

To build this approach, we start with the all-electron charge density in the PAW method for the valence electron bands:

1 $$n\left(r\right)=ñ\left(r\right)+n^{1}\left(r\right)-{ñ}^{1}\left(r\right)$$

where *ñ*(**r**) is the pseudo-charge density, *n* ^1^(**r**) is the all-electron density in the atomic core regions (the superscript 1 refers to on-site terms), and *ñ* ^1^(**r**) is the pseudo-density in these core regions. As the MJ effect is driven by the planewave interactions defined outside of the PAW augmentation spheres, we will focus only on the pseudo-charge density term, *ñ*(**r**).

Underlying this density are the pseudo-wavefunctions within the PAW method, which are expressed as linear combinations of planewaves:

2 $${\mathrm{\psi ̃}}_{k,n}\left(r\right)=\frac{1}{\sqrt{V_{\mathrm{cell}}}}\underset{hkl}{\sum }c_{hkl,k,n}e^{i(G_{hkl}+k)\cdot r}$$

where **G** *_hkl_* = *h* **a** ^*^ +*k* **b** ^*^ +*l* c*, **k** is the location of the wavefunction within the Brillouin zone, *n* is the band index, and *V*_cell_ is the unit cell volume. The full pseudo-charge density, *ñ*(**r**), is then the sum over the bands and *k*-points of the absolute squares of the occupied wavefunctions, expressed as

$$\begin{array}{c} ñ\left(r\right)=\underset{k,n}{\sum }o_{k,n}{|{\mathrm{\psi ̃}}_{k,n}\left(r\right)|}^{2}\\ =\frac{1}{V_{\mathrm{cell}}}\underset{k,n}{\sum }o_{k,n}\underset{hkl}{\sum }\underset{h^{\prime }k^{\prime }l^{\prime }}{\sum }{\;c}_{h^{\prime }k^{\prime }l^{\prime },k,n}^{*}c_{hkl,k,n}e^{i(G_{hkl}-G_{h^{\prime }k^{\prime }l^{\prime }})\cdot r} \end{array}$$ 3

In this way, the full pseudocharge density can be written in terms of contributions from the full range of planewave pairs. Among these planewave pairs are those that contribute to the MJ effect. We can focus in on the structural origins of the MJ effect by going back to the pseudo-wavefunctions underlying this density and applying weighting functions to its planewaves to filter out all but the most relevant contributors, as follows:

4 $${\mathrm{\psi ̃}}_{k,n}^{MJ}\left(r\right)=\frac{1}{\sqrt{V_{\mathrm{cell}}}}\underset{hkl}{\sum }S_{HKL}\left(k,hkl\right)X_{E_{min},E_{max}}c_{hkl,k,n}e^{i(G_{hkl}+k)\cdot r}$$

The first weighting function, *S_HKL_* (**k**,*hkl*), follows the form used in the calculation of MJHPs. It is zero unless (1) there exists a second planewave (**k** + **G**_*h*′*k*′*l*′_) that can interact with the first through the reciprocal lattice vectors of interest (**G**_*h*–*h*′,*k*–*k*′,*l*–*l*′_ = **G** *_HKL_*, a member of a chosen {*HKL*} family of vectors)^69^ and (2) both planewave wavevectors reside within the reciprocal space shell defined by

5 $$\frac{\left|G_{HKL}\right|}{2}-\Delta_{\mathrm{shell}}\leq \left|k+G_{HKL}\right|\leq \frac{\left|G_{HKL}\right|}{2}+\Delta_{\mathrm{shell}}$$

where, Δ_shell_ is an adjustable parameter (set to 0.075 Å^–1^ for all systems) that defines the thickness of the shell in reciprocal space. When both of these conditions are satisfied:

6 $$S_{HKL}\left(k,hkl\right)=e^{-\frac{{\left(|k+G_{hkl}|-|k+G_{h-H,k-K,l-L}|\right)}^{2}}{\sigma }}$$

where, *σ* is set to 0.1 Å^–2^ for all systems (in the Supporting Information, we explore the dependence of the results on the values of the Δ_shell_ and *σ* parameters).

The second weighting function, *X_Emin_*,*_Emax_*, limits the contributions to bands within a defined energy window where the MJ effect is hypothesized to be taking place:

7 $$X_{E_{min},E_{max}}=\{\begin{array}{l} 0,\mathrm{when}{\;\varepsilon }_{k,n}<E_{min}\;or{\;\varepsilon }_{k,n}>E_{max}\\ 1,\mathrm{when}{\;E}_{min}\leq \varepsilon_{k,n}\leq E_{max} \end{array}$$

These windows can be determined simply by reading the energy ranges of the peaks in the MJHP curves as shown below.

By introducing the MJ-weighted wavefunctions into eq 3, we arrive at an electron density associated with the MJ effect,

8 $${ñ}^{MJ}\left(r\right)=\underset{k,n}{\sum }{|{\mathrm{\psi ̃}}_{k,n}^{MJ}\left(r\right)|}^{2}$$

which can be visualized using isosurface plots.

The *ñ* ^*MJ*^(**r**) represents the electron density from a basis set of planewaves that satisfy the conditions of the weighting functions in eq 4. It is dominated by pairs of planewaves that are interacting through the reciprocal lattice vector of interest, **G** *_HKL_*, in the wavefunctions over a selected energy window. At the same time, it also incorporates all the other minor couplings among the planewaves in this select basis set in these wavefunctions.^70^ To return to the full pseudo-charge density, one need only add the contributions from the pairs of planewaves both excluded by the weighting functions, *ñ* ^remainder^(**r**), and pairs in which an excluded planewave is combined with an included one, *ñ* ^overlap^(**r**), leading to

9 $$ñ\left(r\right)={ñ}^{MJ}\left(r\right)+{ñ}^{\mathrm{remainder}}\left(r\right)+{ñ}^{\mathrm{overlap}}\left(r\right)$$

As we will see in the next sections, an examination of the *ñ^MJ^* (**r**) functions for a variety of intermetallic structures derived from the BCC arrangement reveals a surprisingly simple structural mechanism for supporting the MJ effect.

### 3.2 CuZn (β-Brass, CsCl Type)

For our first illustration of the Mott–Jones electron density, let's consider β-brass, CuZn, which adopts the simple CsCl structure type. This arrangement is an ordered variant of the body-centered cubic (BCC) structure with different colorings of the body-center (Zn) and corner (Cu) points in the cell. In this way, it represents a 1 × 1 × 1 BCC supercell, in which the *I*-centering of the original BCC cell has given way to *P*-centering. The overall valence electron concentration for this compound is 1.5 electrons/atom (when we consider each Cu and Zn atom as bringing 1 and 2 valence electrons to the system, respectively).

In Figure 3a, we highlight the influence of the MJ effect on the electronic structure of this compound, beginning with a plot of its DOS distribution (left), following our earlier MJHP analyses.^49,66^ Here, a broad pseudogap in the DOS is seen to straddle the Fermi energy (*E*_F_). The *MJHP_HKL_* (*E*) curve for the {110} vectors (right) confirms the MJ effect with a transition between stabilizing (blue) and destabilizing (red) contributions coinciding with the pseudogap. The integrated *MJHP_HKL_* (*iMJHP_HKL_*) value up to the *E*_F_ is −347.1 meV/atom.

**Figure 3:**
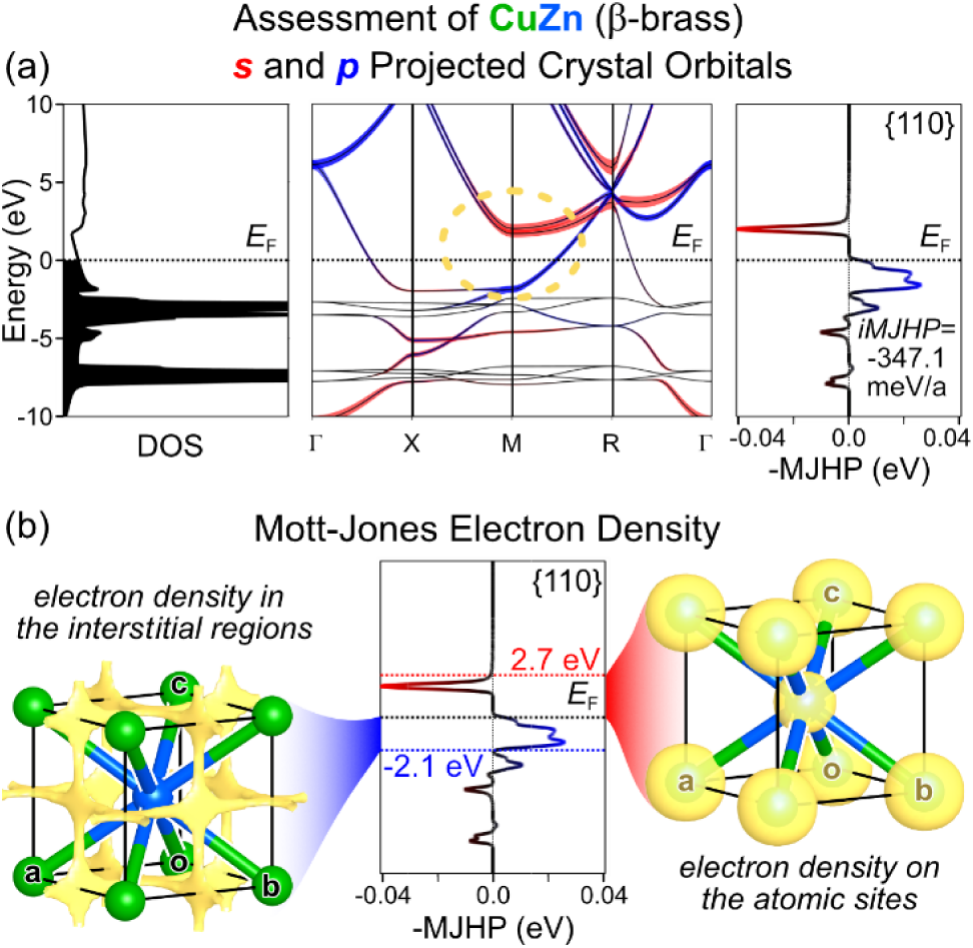
Connection between MJ interactions and electron density in β-brass, CuZn. (a) The band structure of CuZn shown with *s-* and *p-*type contributions highlighted using a fat-band representation, shown in relation to the DOS distribution and *MJHP*_HKL_(*E*) plot to the left and right, respectively. In this *MJHP_HKL_* (*E*) plot and those that follow, red and blue indicate destabilizing and stabilizing energetic contributions. (b) The MJ electron density obtained for {*HKL*} = {110} in the stabilizing energy range, −2.1 to 0.0 eV (left), and the destabilizing energy range, 0.0 to 2.7 eV (right).

Using the MJ electron density, we can see how the atomic positions within the structure contribute to this effect. In Figure 3b, we plot the MJ electron density derived from the {110} family of planewaves in CuZn for two different energy ranges, corresponding to the stabilizing (left) and destabilizing (right) contributions in the *MJHP_HKL_* (*E*) curve. They tell two distinct stories. The Mott–Jones electron density derived from bands in the destabilizing energy range, from 0 to 2.7 eV above the *E*_F_, simply shows maxima directly on the atomic positions. By contrast, in the stabilizing energy window, from −2.1 eV to the *E*_F_, the MJ electron density resides in the interstitial regions. Each of the structure’s compressed octahedral holes is centered by a density maximum, with the density being flattened along the compressed direction and connected in a square planar fashion to its counterparts in neighboring holes along the perpendicular directions.

How do these density features connect to the observed splitting of stabilizing and destabilizing interactions? As the density maps are created from a small number of planewaves, the minima in the density will generally coincide with nodal planes in the standing waves they create. The concentration of electron density on the atom centers in the higher-energy case then arises from nodes that pass between the atoms but not through them. The overall wavefunctions would then resemble arrays of *s*-orbitals that are out-of-phase with each other, i.e., *antibonding* combinations of *s*-orbitals. Meanwhile, the lower-energy case’s accumulation of electron density in spaces directly between atoms could be derived from summed density arising from *bonding* combinations of *p*-orbitals directed along *x*, *y*, and *z*. The splitting can be interpreted then in terms of the gap between *p*–*p* bonding and *s*–*s* antibonding interactions, as envisioned by Berger and coworkers,^9^ which are templated by the nodal properties of the standing waves.

Evidence for this scheme is discernible in the projections of the wavefunctions onto *s*- and *p*-characters, as shown with a fat-band representation in Figure 3a. Here, the relative *s-* and *p*-orbital contributions are shown with the thicknesses of the bands using red and blue, respectively. With a dotted circle, we direct our attention to the *M* point (0.5, 0.5, 0.0), where the couplings of the {110} family of reciprocal lattice vectors are predicted to be strong by the MJ picture. Around 2 eV below *E*_F_ at the *M*-point, there is a pair of nearly degenerate bands with strong *p*-type contributions. Similarly, about 2 eV above *E*_F_ at the *M*-point, there exists a pair of nearly degenerate bands with a large *s*-type character. These crystal orbitals below and above *E*_F_ align energetically with the stabilizing and destabilizing contributions of the {110} family of reciprocal lattice vectors, respectively. It should be noted, though, that *s*- and *p*-orbital characters are not always associated with {110} MJ interactions, as can be seen by the red and blue colorings of bands outside of the dotted yellow circle.

The assignment of *p*- and *s*-type orbital character to stabilizing and destabilizing MJ interactions is also consistent with the shape of the *MJHP_HKL_* (*E*) curve. Note that the locations of the flat bands with strong *s-* or *p*-orbital contributions below and above the *E*_F_ at the *M*-point correspond to the sharp peaks of the *MJHP_HKL_* (*E*) curve. Meanwhile, the steeply sloped bands along the path from *M* to *R* produce the broader *MJHP_HKL_* (*E*) features, where the *p*-character is maintained along the path. Although the pair of bands above the *E*_F_ from *M* toward *X* possess a similarly steep slope, there is little *s*-orbital contribution and hence no broadening of the destabilizing peak in the *MJHP_HKL_* (*E*) curve.

From this analysis, a straightforward connection emerges between the {110} family of reciprocal lattice vectors and the atomic arrangement of Cu–Zn. We begin with a free-electron system and inscribe its Fermi sphere with a cuboctahedron of points, corresponding to the {110} family of reciprocal lattice vectors of a cubic lattice. Turning on a potential with Fourier components at these points induces a mixing of planewaves on the Fermi sphere. The results are standing waves, which give rise to two types of density maps: a BCC arrangement of density peaks and its complement, where the density populates the interstices of the BCC arrangement. These density maps, in turn, provide a template for atomic positions, in which the placement of atoms between density maxima allows them to supplement the standing waves that underlie the densities with *p*–*p* bonding. As we move from CuZn to more complex intermetallics exhibiting MJ stabilization, we will see that this picture is conserved to a remarkable degree.

### 3.3 2 × 2 × 2 BCC Supercells: BiF_3_-Type AlCu_3_ and CaF_2_-Type AuAl_2_

Let's now move to some slightly more complicated structures stabilized by the MJ effect: 2 × 2 × 2 supercells of the BCC arrangement. Our first example is AlCu_3_,^53^ which adopts the BiF_3_ type, a binary variant of the AlCu_2_Mn-type Heusler structure. Its structure can be derived by considering a BCC arrangement of Cu atoms, taking a 2 × 2 × 2 supercell, and then replacing every other corner atom of the subcells with an Al atom (or, alternatively, replacing the atoms at the corners and face centers of the supercell with Al).

The MJ effect plays an active role in the electronic stability of AlCu_3_. If we consider the Al and Cu atoms to each contribute 3 and 1 valence electrons to the system, respectively, then we obtain an electron concentration identical to that of CuZn, 1.5 electrons/atom. The diameter of the Fermi sphere for a free electron system with this electron density matches the diffraction angle for the {220} family of reciprocal lattice vectors of AlCu_3_ (see the Supporting Information). Indeed, as seen in Figure 4a, a broad pseudogap in the DOS distribution at the *E*_F_ coincides in energy with the stabilizing and destabilizing potential energy contributions from the {220} family of reciprocal lattice vectors (*iMJHP*_220_ = −364.4 meV/atom).

**Figure 4:**
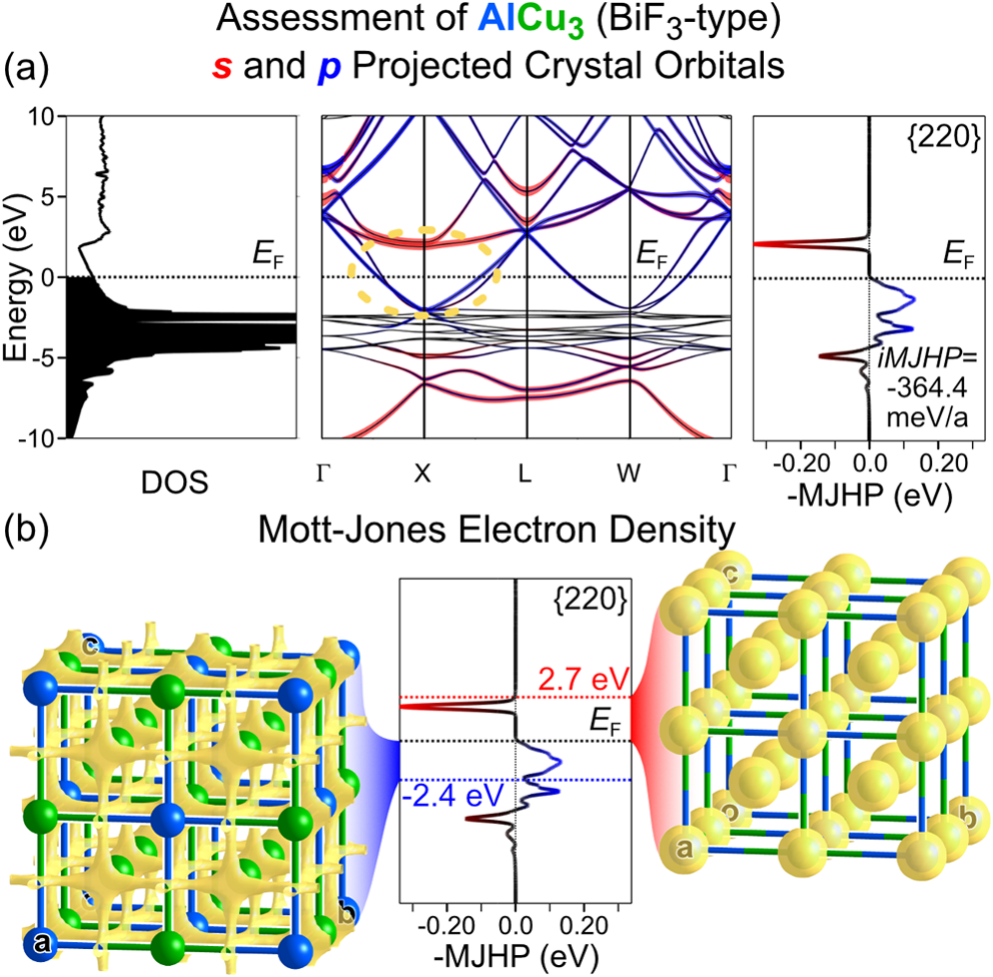
MJ electron density analysis of AlCu_3_ (BiF_3_ type). (a) DOS distribution, *s*-/*p*-orbital weighted band structure, and *MJHP*_{220}_(*E*) curve. (b) The MJ electron densities for the {220} vectors derived from bands in the stabilizing energy range (−2.4 to 0.0 eV, left) and the destabilizing energy range (0.0 to 2.7 eV, right).

In Figure 4b, we show the corresponding MJ electron densities for AlCu_3_. In the stabilizing energetic window below the *E*_F_, the MJ electron density resides in the interstitial regions, which is nearly identical to that obtained for CuZn. Its concentration of density along the interatomic contacts is again suggestive of *p*–*p* bonding interactions. Above the *E*_F_, in the destabilizing energy range, the electron density lies directly on the atomic sites filling a 2 × 2 × 2 BCC network, indicative of *s*–*s* antibonding interactions.

The *s-* and *p-*contributions to the wavefunctions, overlaid on the band structure in Figure 4a (middle), confirm these conclusions. Here, we focus on the special point *X* (0.5, 0.5, 0.0) in the primitive cell (which would be folded to Γ if the conventional cell were used in the calculation), where the {220} family of conventional reciprocal lattice vectors ({112} in the primitive setting) are expected to couple the planewaves on the hypothetical free electron Fermi surface most strongly. At the *X*-point, the *E*_F_ is framed by pairs of crystal orbitals above and below that possess strong *s*-orbital and *p*-orbital contributions, respectively. Like CuZn, the dominant features of the *MJHP_HKL_* (*E*) curve align with the slopes of the bands with strong *s* or *p* character.

The MJ density features and their correlation with stabilizing and destabilizing interactions are essentially indistinguishable from those of CuZn. In fact, all that has really changed is that the vectors we labeled {110} in CuZn are now called {220}, reflecting that the site coloring in AlCu_3_ has created a doubling of the cell along the *a*, *b*, and *c* directions. The overall stabilization mechanism is unchanged by this variation in elemental identities.

Similar results are obtained for another 2 × 2 × 2 supercell of the BCC structure: AuAl_2_,^71^ which adopts the CaF_2_ structure type (Figure 2c), a binary variant of the MgAgAs-type half-Heusler structure.^72^ To derive its structure, we start with a CsCl-type AuAl phase and remove every other Au atom to create a 2 × 2 × 2 supercell. The overall valence electron concentration for this compound is 2.33 or 7/3 electrons/atom (considering Au and Al as contributing 1 and 3 valence electrons, respectively), which is relatively high for a BCC-derived structure. However, if we instead include the vacancies in the atom count (contributing 0 electrons), we obtain 1.75 electrons/site, in the expected range for a BCC superstructure.

The validity of this scheme is apparent in a MJHP analysis of AuAl_2_, as shown in Figure 5a. Even as *E*_F_ falls below the nearest DOS pseudogap (which in fact corresponds to the filling of an 18-electron configuration at one extra electron per formula unit),^73^ it does align with a transition between stabilizing and destabilizing contributions from the {220} family of reciprocal lattice vectors, with the *iMJHP*_220_ value being −214.5 meV/atom.

**Figure 5:**
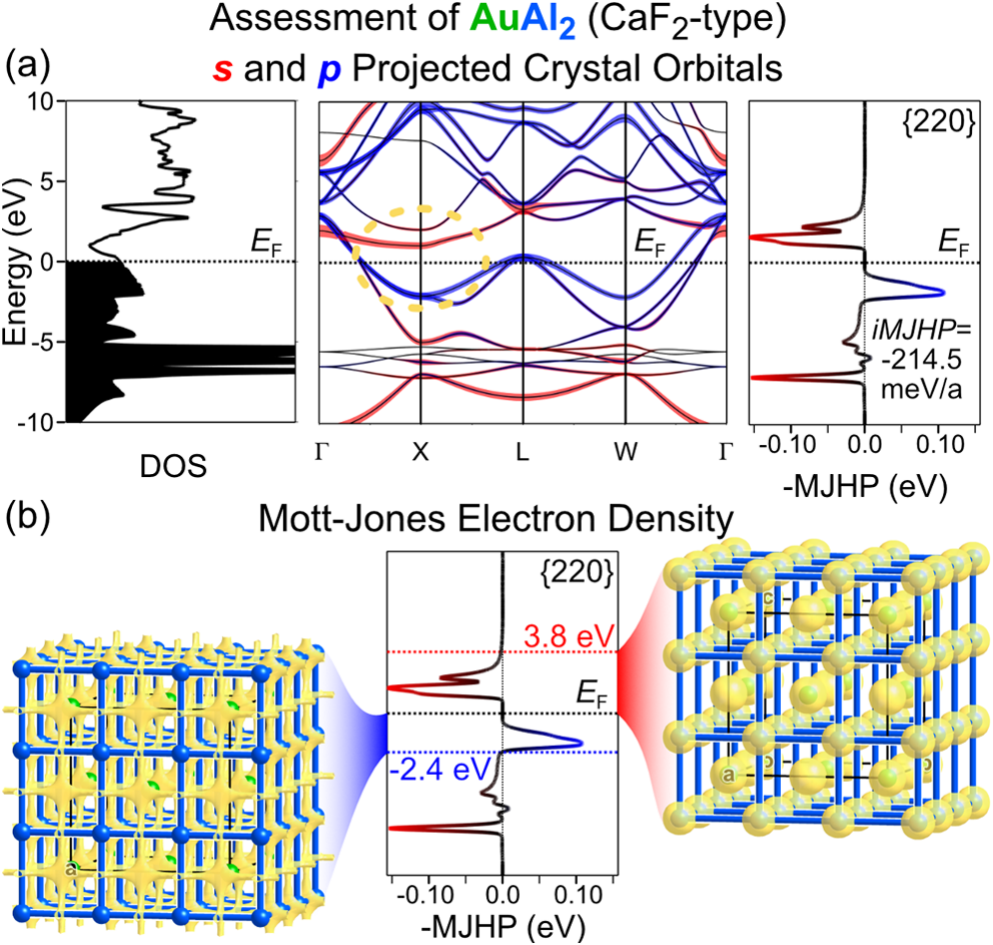
MJ electron densities in AuAl_2_ (CaF_2_ type). (a) Alignment of the DOS features, *s-* and *p-*orbital contributions to bands at the *X-*point, and *MJHP*_{220}_(*E*) peaks. (b) The MJ electron densities for the {220} vectors derived from bands in the stabilizing energy range (−2.4 to 0.0 eV, left) and the destabilizing energy range (0.0 to 3.8 eV, right).

The MJ electron density associated with these stabilizing and destabilizing MJHP peaks (Figure 5b) reveals motifs similar to those we observed for CuZn and AlCu_3_. Above the *E*_F_, the MJ electron density features form a BCC lattice, while its inverse is found for the MJ density below the *E*_F_, irrespective of the missing atoms relative to a BCC/CsCl-type arrangement. The electron density pattern is unperturbed by the vacant Au sites, illustrating that the free-electron character of the crystal orbitals near the *E*_F_ simply ignores defects.

The *s*/*p*-contribution-weighted band structure (Figure 5a) for AuAl_2_ affirms its similarities to the systems we examined earlier. At the *X* point, *p*-orbital contributions dominate in a pair of degenerate crystal orbitals below the *E*_F_, which are aligned with the stabilizing potential energy contributions in the *MJHP_HKL_* (*E*) curve. Above the *E*_F_, meanwhile, there are *s*-orbital contributions that match the strong destabilizing interactions in the *MJHP_HKL_* (*E*) plot.

### 3.4 γ-Brass, Cu_5_Zn_8_, a 3 × 3 × 3 BCC Superstructure

From our examination of the 1 × 1 × 1 and 2 × 2 × 2 superstructures, a general picture has emerged: the strongest Fourier components in the potential seed the formation of electron density maps corresponding to a BCC array and its inverse. The atoms within the structure are placed on the peaks of one map (and thus the interstices of the other), leading to an energy splitting between them and the formation of a pseudogap. The actual indices for the reciprocal space vectors involved depend on the size of the BCC supercell that emerges from site coloring and vacancy distributions.

With this picture in hand, we now move to a more complex case in which the connections to the BCC structure are more distant: Cu_5_Zn_8_, also known as γ-brass. The structure of Cu_5_Zn_8_ (Figure 2d) is usually visualized as a body-centered packing of characteristic units referred to as γ-brass clusters. A classic description of these structures is in terms of concentric polyhedra created by the orbits of the symmetry-distinct sites: an inner tetrahedron at the center, followed by an outer tetrahedron, octahedron, and cuboctahedron.^74^ In Figure 2, we take an alternative approach to highlight a connection to the MgCu_2_-type Laves phase structure: the Cu atoms trace out an adamantane-like cage, which is interpenetrated by a tetrahedron of vertex-sharing tetrahedra of Zn atoms.^75^

Less obvious from either depiction of this structure is its well-known connection to the BCC structure.^55^ It can be built from a 3 × 3 × 3 BCC supercell, removing the atoms at the supercell corner and center, and allowing the structure to relax. The atoms in each vacant cube move alternately inward toward and outward from the vacancy to form stella quadrangula. How does the resulting 3D configuration of atoms contribute to the MJ interactions long attributed to the γ-brass structure?

MJHP analysis confirms the presence of these interactions. The electronic DOS distribution calculated for the phase exhibits a deep minimum near the *E*_F_ (Figure 6a). This pseudogap aligns with two different pairs of stabilizing and destabilizing potential energy contributions in the *MJHP*(2θ,*E*) analysis (as provided in the Supporting Information), whose positions match the diameter of the hypothetical Fermi sphere for Cu_5_Zn_8_’s valence electron concentration of 21/13 or 1.615 electrons/atom. These pairs of peaks arise from the {330} and {±411} families of reciprocal lattice vectors, with the {330} contributing a higher magnitude (*iMJHP_HKL_* = −266.0 meV/atom) compared to the identical {411} and {4̅11} vectors (*iMJHP_HKL_* = −112.7 meV/atom).

**Figure 6:**
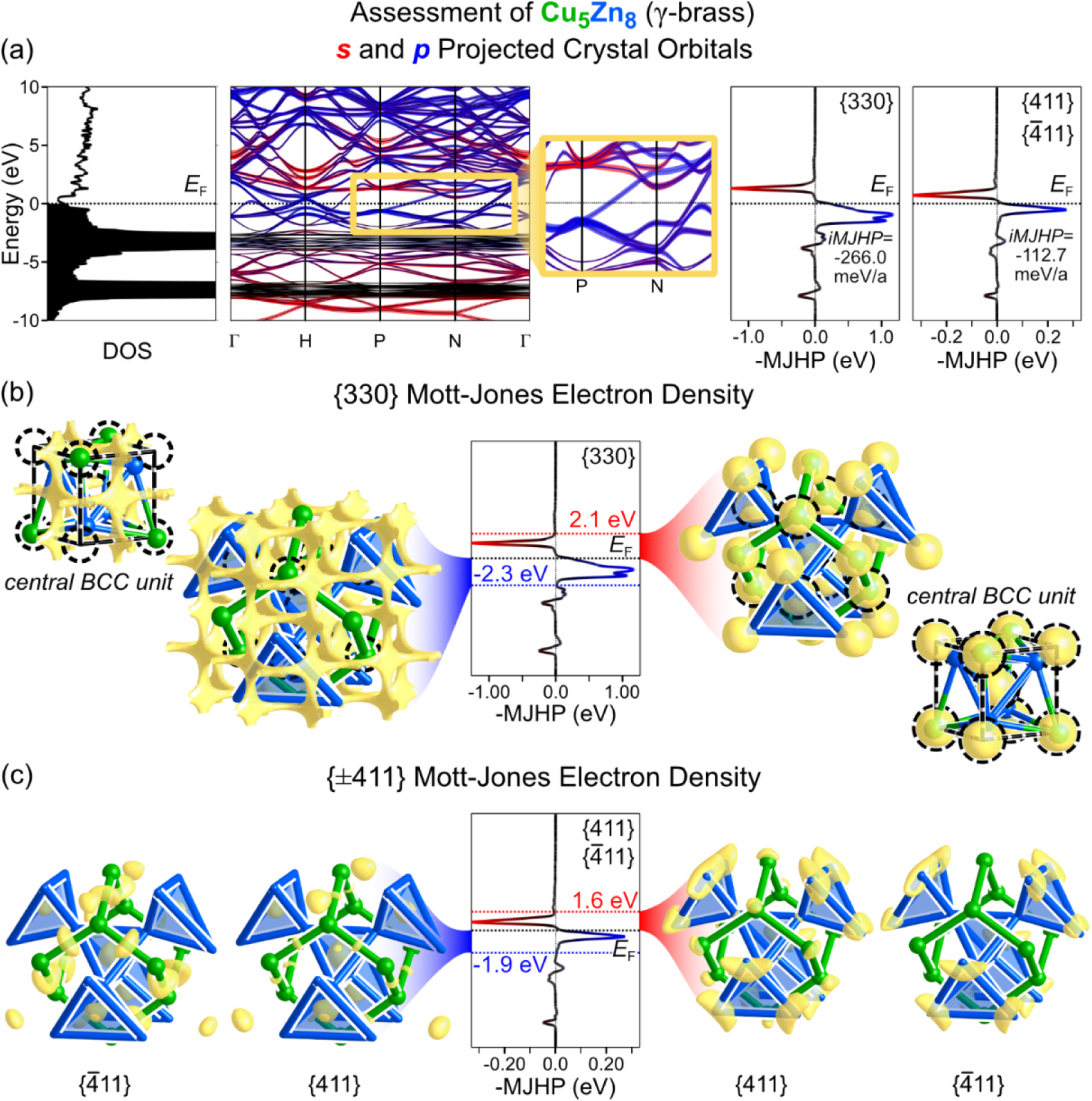
MJ electron densities in γ-brass, Cu_5_Zn_8_. (a) Electronic DOS distribution, *s-* and *p-*orbital weighted band structure, and the *MJHP_HKL_* (*E*) curves for the {330} and {±411} reciprocal lattice vectors. (b) MJ electron densities plotted for one γ-brass cluster (see the Supporting Information for the full unit cell), shown for the {330} vectors in the stabilizing and destabilizing energetic windows on the left and right, respectively. (c) Corresponding densities for the {4̅11} and {411} reciprocal lattice vectors. In (b), a close-up of the central stella quadrangula is shown to highlight the relationship of the displacements from an idealized cube and the density features.

Using MJ electron density maps, we can correlate these stabilizing interactions to the features of Cu_5_Zn_8_’s structure. In Figure 6b, we present the maps for the {330} family of vectors, focusing for simplicity on one of the cell’s two γ-brass clusters (identical maps are obtained for the second cluster, as may be seen in the Supporting Information). Despite the increased complexity of Cu_5_Zn_8_ compared to the previous BCC supercells, the same dominant features appear. Below the *E*_F_ in the stabilizing energy range, the electron density traces out a now familiar path along the interstitial regions of a simple BCC structure, even as the atoms themselves no longer adopt this idealized arrangement.

Intriguingly, the displacements of the atoms from the BCC arrangement have a clear relationship to these density features, as we highlight with a close-up of the cluster’s central stella quadrangula on the far-left hand side of Figure 6b. The stella quadrangula’s inner tetrahedron is formed by Zn atoms contracting through the channels of minimal MJ electron density. When we recall that these channels are formed from the nodal surfaces of standing waves, it becomes evident that this motion has maintained the opportunity for the atoms to contribute *p*-orbitals to the crystal orbitals. As such, continuity is maintained with the bonding situation of the simpler BCC derivatives.

In terms of the destabilizing energetic window above the *E*_F_, the MJ electron density occupies the lattice positions of a 3 × 3 × 3 BCC supercell, seemingly oblivious to the absence or misalignment of the atoms. The proximity of the remaining atoms to density maxima suggests that they are collectively poised to contribute antibonding combinations of *s*-orbitals to the wavefunctions.

We confirm this hypothesis through an inspection of the orbital-resolved band structure (Figure 6a), paying particular attention to the *N*-point (0.0, 0.0, 0.5 when using the primitive cell, 0.5, 0.5, 0.0 for the conventional cell) where the {330} family of reciprocal lattice vectors ({003} in the primitive cell) are expected to couple planewaves the most strongly, and the neighboring *P*-point (0.25, 0.25, 0.25 for the primitive cell; 0.5, 0.5, 0.5 for the conventional cell) where mixings from the {330} family of reciprocal lattice vectors may also occur. About 3 meV below the *E*_F_, a steep set of bands dominated by *p*-orbital contributions runs between these points, aligning with the stabilizing potential energy splitting in the *MJHP_HKL_* (*E*) curve. About the same distance above the *E*_F_ at the *P*-point, there is a relatively flat pair of crystal orbitals with strong *s*-orbital weights, which fade to *p*-orbital character as the *N*-point is approached. As in the cases we saw earlier, at the special *k*-points most associated with MJ interactions, a large energy gap about the *E*_F_ correlates with the splitting of *s*- and *p*-based crystal orbitals.

A new feature in Cu_5_Zn_8_ relative to the simpler BCC-derivatives is the emergence of MJ interactions at reciprocal lattice vectors beyond the {*HH*0} set. The {411} and {4̅11} vectors are also involved in MJ interactions, though with lower strengths. The MJ electron density associated with these vectors (Figure 6c) tells of an effect tailored to the compound’s unique geometrical features. In the stabilizing energy window, below the *E*_F_, the MJ electron density resides in two distinct regions: the interstices of the vertex-sharing Zn tetrahedra and the midpoints of Cu–Cu contacts within the adamantane cage.^76^ As for the {330} case, the atomic positions lie conspicuously in the openings of this density.

Above the *E*_F_, in the destabilizing energy range, the MJ electron density piles onto nonspherical lobes that are somewhat displaced from the atomic centers. Curiously, though, rather than reflecting the mismatch between the atomic positions and the BCC parent structures, these electron density features are placed to represent exaggerations of the ways the atoms have shifted. This distribution of density suggests dominant *s*-orbital effects with hybridization. These expectations are confirmed by the angular momentum-resolved crystal orbital diagram, where we are again interested in the *P-* and *N*-points where the {411} and {4̅11} ({1̅22} and {32̅2̅} in the primitive cell, respectively, that fold onto the *N*-point) families are expected to couple the pairs of planewaves on the hypothetical Fermi sphere the strongest. Directly under the *E*_F_ along the path from the *N*-point to the Γ-point, there is a pair of bands with strong *p*-orbital contributions that coincide directly with the stabilizing potential energy peak in the *MJHP_HKL_* (*E*) curve for the {411} and {4̅11} families. Directly at the *N*-point and above the *E*_F_, we can identify a pair of bands with both *s-* and *p*-orbital weights that align with the destabilizing splitting in the *MJHP_HKL_* (*E*) plots.

Sums of the MJ electron densities for the {330} and {411}/{4̅11} vectors bring the role of the latter vectors into focus (Figure 7). In the stabilizing energy range, including the {411}/{4̅11} density shifts the density maxima from the {330} map toward the centers of the interstices of Cu_5_Zn_8_. Similarly, in the destabilizing range, the {411}/{4̅11} density subtly tunes the positions of the density features for the {330} to better match the atomic positions of Cu_5_Zn_8_. Despite the slight variations highlighted here, the sums of the density maps from the {330}, {411}, and {4̅11} vectors largely resemble the original {330} MJ electron densities, particularly for the range above the *E*_F_. This emphasizes the dominance of the {330} family of reciprocal lattice vectors. Altogether, the {411}/{4̅11} MJ interactions can be viewed as a modulation of the MJ electron density derived from the BCC parent structure.

**Figure 7:**
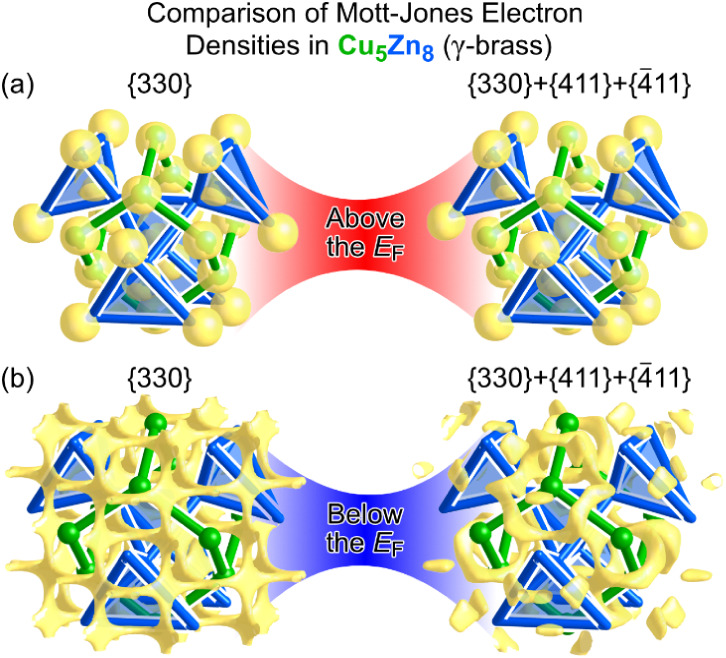
Comparison of the MJ electron densities for the {330} (left) and summed {330}, {411}, and {4̅11} (right) families of reciprocal lattice vectors in Cu_5_Zn_8_ (γ-brass) in their respective energy ranges (a) above the *E*_F_ and (b) below the *E*_F_.

The results are combined MJ electron densities corresponding to (1) interstices and (2) atomic centers, all formed from planewaves near the Fermi surface of a free electron gas. In the absence of atomic potentials, these density maps would be essentially degenerate, as they are formed from noninteracting planewaves of nearly the same energy. Turning on the atomic potentials, however, opens an energy gap between the two.

### 3.5 The 4 × 4 × 4 BCC Supercell: Ga_4_Ni_3_-Type Al_4_Ni_3_

We now arrive at the final and largest BCC supercell in Figure 2e, Al_4_Ni_3_ (Ga_4_Ni_3_ type).^57^ Its structure may be understood in terms of 4 × 4 × 4 supercell of a CsCl-type AlNi phase, with the Al atoms adopting a primitive cubic lattice and the Ni atoms occupying the cubic holes. The Al_4_Ni_3_ structure is formed from this arrangement by removing one out of every four Ni atoms. The electronic relationship to the BCC derivatives we’ve already examined is evident in its valence electron concentration of 1.71 electrons/atom (considering each Ni and Al atom as bringing 0 and 3 electrons, respectively) or 1.5 electrons/site in the BCC parent structure.

The electronic DOS distribution for Al_4_Ni_3_ (Figure 8a) features a noticeable pseudogap around 1.6 eV above the *E*_F_. Reaching this minimum would require around 3 additional electrons per formula unit. Despite the absence of a well-defined DOS opening at the *E*_F_, the *MJHP*(2θ,*E*) analysis (given in the Supporting Information) reveals a strong pair of potential energy contributions at a 2θ angle coinciding with the hypothetical free-electron Fermi sphere diameter. As expected from our experiences with the earlier systems, these peaks correspond to the {440} family of reciprocal lattice vectors. The strength of this interaction is on the same order as those we have seen above, with *iMJHP_HKL_* = −144.6 meV/atom.

**Figure 8:**
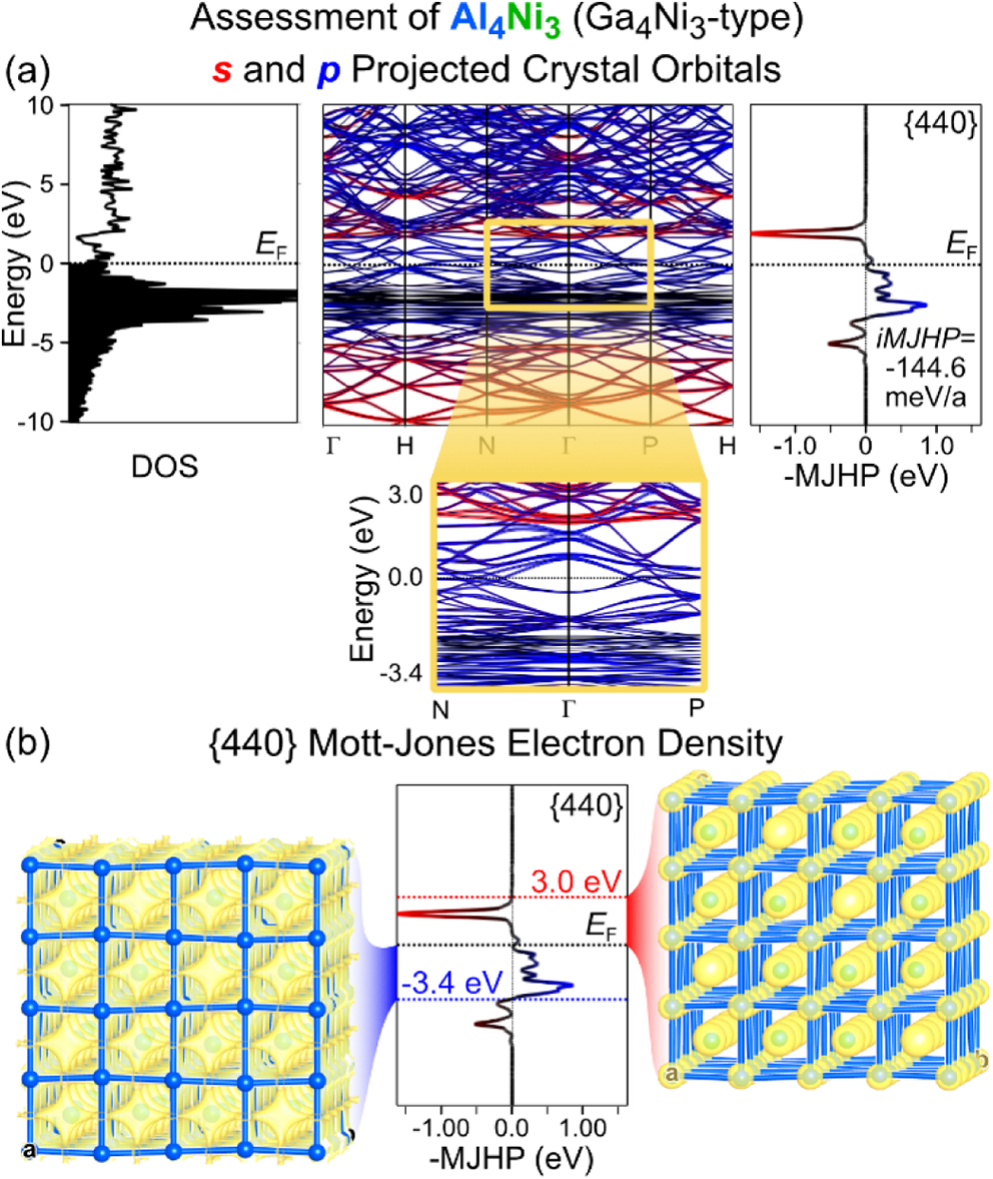
MJ electron densities in Ga_4_Ni_3_-type, Al_4_Ni_3_. (a) Electronic DOS distribution, *s-* and *p-*orbital weighted band structure, and the *MJHP_HKL_* (*E*) curves for the {440} reciprocal lattice vectors. (b) The MJ electron densities for the {440} vectors derived from bands in the stabilizing (left) and destabilizing (right) energy range.

The MJ electron density for the {440} vectors in Figure 8b shows the characteristic patterns we have seen for other BCC superstructures. In the stabilizing energy range, below the *E*_F_, the electron density is situated between the BCC lattice sites, indicative of *p*-orbital contributions. Above the *E*_F_, in the destabilizing window of energy, the electron density lies directly on the lattice positions corresponding to a simple 4 × 4 × 4 BCC supercell, in an *s*-orbital-like arrangement. This analysis is confirmed through inspection of the fat bands of Figure 8a, where we focus primarily on the Γ-point (0.0, 0.0, 0.0), where the {440} ({004} in the primitive cell) family of reciprocal lattice vectors are expected to couple the pairs of planewaves on the Fermi surface the most strongly. Around 2.5 eV below the *E*_F_ at the Γ-point, there is a bundle of crystal orbitals with significant *p*-orbital contributions that coincides with the stabilizing potential energy peaks in the *MJHP*_{440}_(*E*) curve. Conversely, around 1.5 eV above the *E*_F_ at Γ, there is a set of bands with strong *s*-orbital weights. Although the increased cell size of this compound adds a layer of complexity, the MJHP analysis clearly ties the MJ effects to the underlying BCC parent structure.

Within the energy gap between the {440} contributions, we also discern in the *MJHP*(2θ,*E*) plot a weaker pair of stabilizing and destabilizing peaks split across *E*_F_. These peaks originate from the {215} reciprocal lattice vectors and make minor energetic contributions to the bonding (see the Supporting Information for further details).

### 3.6 The Mott–Jones Electron Crystal

From the five examples explored in Sections 3.2–3.5, a more general picture for the real-space implications of the MJ effect comes into view. These BCC superstructures are united by the presence of strong MJ interactions associated with their {*HH*0} reciprocal lattice vectors. Whether *H* is 1, 2, 3, or 4, these reciprocal lattice vectors form a constellation around Γ in the shape of a cuboctahedron. It would seem that the cuboctahedral shape is more important to the form of the MJ electron density maps than the specific indices we assign to the vertices. As an illustration of this point, we show in Figure 9a maps created by combining the planewaves whose wavevectors come from opposite corners of a cuboctahedron into cosine and sine functions, squaring them, and adding the cosine and sine series separately to form hypothetical MJ electron densities. The results are nearly indistinguishable from those we derived from the DFT pseudo-wavefunctions, with the sine-based map corresponding to the stabilizing energy windows and the cosine-based ones to the destabilizing windows.

**Figure 9:**
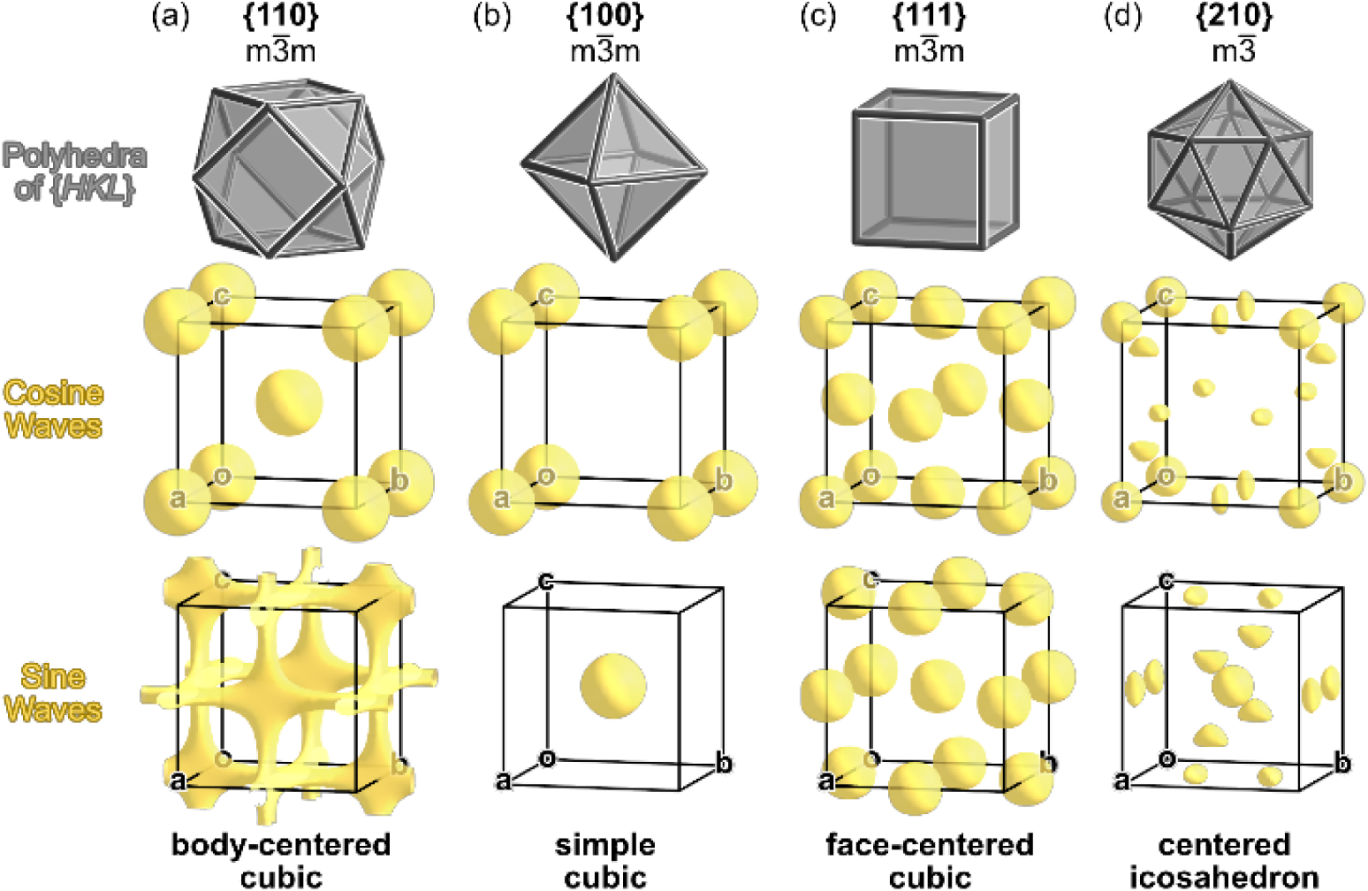
Hypothetical MJ electron densities formed from the cosine (middle) and sine (bottom) combinations of planewaves coupled through the (a) {110}, (b) {100}, (c) {111}, and (d) {210} families of reciprocal lattice vectors, considering *m*3̅*m* and *m*3̅ symmetry for (a)-(c) and (d), respectively. The polyhedra traced out by the combination of {*HKL*} vertices are shown in the top panel.

Intriguingly, as we have seen for the structures with site vacancies and distortions away from the BCC arrangement, the MJ electron density features associated with the {*HH*0} reciprocal lattice vectors are invariant with respect to the specific details of atomic placement in the structure. Instead, they provide a template for such positions, with the gaps in the interstitial map suggesting channels along which atoms may move without seriously disrupting the MJ interactions. Inspired by this partial decoupling of the electronic and nuclear degrees of freedom, the long-range ordered pattern created by the MJ electron density may be interpreted as a *Mott–Jones electron crystal*, which resembles a charge-density wave^50,51^ with simultaneous nesting of the Fermi surface along multiple directions. However, while a charge-density wave involves the modulation of an existing arrangement of atoms, a Mott–Jones electron crystal sets up the atomic arrangement itself.

The cuboctahedron defined by the {*HH*0} vectors in the systems studied here represents just one of an infinite number of ways that the surface of a free-electron Fermi sphere might be decorated with reciprocal space vectors corresponding to the Fourier components of a potential. In Figure 9b–d, we demonstrate that other configurations with cubic symmetry can give rise to different MJ electron crystals. The {100} family of vectors (Figure 9b) would inscribe a Fermi surface in an octahedron. The corresponding sine- and cosine-based MJ densities correspond to primitive cubic arrays of density offset from each other by a shift of (1/2, 1/2, 1/2).

A placement of atoms into a selection of the density peaks of one map (and thus interstices of the other) would create a splitting of the energies of these two potential MJ electron crystals. Indeed, preliminary calculations with the norm-conserving pseudopotentials suggest that this is realized in MgCu_2_-type ZrZn_2_, where the optimization of a {400} MJ interaction is correlated with a MJ electron density that follows a 4 × 4 × 4 supercell of the cubic primitive array with density peaks in the stabilizing energy window lying in interstitial spaces (the density in the destabilizing window is complicated but involves a concentration of electrons on the Zn atoms).

Likewise, the {111} family of vectors traces out a cube in reciprocal space. The corresponding MJ electron densities define two face-centered cubic arrays of density, staggered so that the density maxima of one lie in the octahedral holes of the other (Figure 9C). The AuCu_3_-type structures, such as AlNi_3_, are poised to take advantage of this scheme. Indeed, an MJHP analysis of AlNi_3_ shows strong MJ interactions associated with the {111} vectors.^66^

Finally, we consider a more complicated pattern, this time in *T*_*h*_ symmetry: the icosahedron whose vertices are somewhat displaced to allow the simple integer indices of {210} with *m*3̅ point symmetry. The resulting MJ electron densities consist of icosahedra inscribed in a cubic cell, rotated by 90° relative to each other, with an additional peak in either the cell corner or center. The emergence of near-icosahedral features highlights the diversity of atomic arrangements that could give rise to MJ stabilization. One deciding factor among the various possibilities may be the requirement that the population of atomic positions templated by the MJ electron crystal leads to reasonable interatomic distances.

## 4 Conclusions

The Mott–Jones (MJ) effect is one of the main mechanisms for the emergence of electronic pseudogaps in intermetallic phases, but its use in materials design is limited by a fundamental incongruity. It is generally regarded as a reciprocal space phenomenon, in which the matching of vectors in *k*-space between electronic and nuclear periodicities creates opportunities for new interactions. At the same time, however, it drives the formation of alluring 3D atomic arrangements—most notably the γ-brass clusters of Cu_5_Zn_8_. In this article, we have attempted to bridge this conceptual divide through the definition of the MJ electron density. Here, we focus on the crystal orbitals in the energy regions corresponding to stabilizing or destabilizing MJ interactions, as identified in a MJ Hamilton Population analysis, and reduce them to their planewave contributors that are active in the MJ effect for a family of reciprocal lattice vectors. Two MJ electron densities arise from the corresponding partial wavefunctions, for the stabilizing and destabilizing energy windows. These are complementary in the sense that together they provide a basis for the creation of a homogeneous electron distribution but separately represent potential interstitial and atomic regions.

In conjunction with the Mott–Jones Hamilton Population analysis, we applied this MJ electron density approach to a series of compounds adopting *H* × *H* × *H* supercells of a BCC parent structure: CsCl-type CuZn (β-brass), BiF_3_-type AlCu_3_, CaF_2_-type AuAl_2_, Cu_5_Zn_8_ (γ-brass), and Ga_4_Ni_3_-type Al_4_Ni_3_. A common trend emerged. Strong MJ effects from the {*HH0*} family of reciprocal lattice vectors were identified in each case. The MJ electron density associated with stabilizing planewave interactions trace out the interstitial regions of a BCC subcell, a situation synergistic with *p*–*p* bonding interactions. In contrast, the destabilizing planewave interactions pile electron density directly onto atomic positions in the BCC parent structure, a scenario that recalls *s*–*s* antibonding. The net result is an opening in the band structure that separates *s-* and *p*-orbital character.

The electron density features from the {*HH0*} family of vectors are largely invariant with respect to the different colorings and vacancies in the example systems. We refer this MJ electron density derived from the {*HH0*} vectors, which trace out a cuboctahedron of vertices around Γ, as the *Mott–Jones electron crystal*. As an extension of this idea, we mapped combinations of planewaves from other polyhedra, whose vertices are given by cubic combinations of {*HKL*} indices, into a series of cosine- and sine- based MJ densities, where we found electron density templates corresponding to packings common to intermetallic systems.

We are also looking forward to exploring new possibilities suggested by the concept of the MJ electron crystal. First, in the γ-brass structure, we saw how the low-density channels of a MJ electron crystal can provide paths for atoms to shift in response to vacancies within a structure. It will be interesting to see whether similar structural effects arise with other MJ electron crystals than those which are derived from the BCC parent structure. Second, in all of the cases we have considered here, the reciprocal space vectors involved in MJ effects have integer indices with respect to linearly independent basis vectors. Such integers imply the presence of an underlying unit cell. An important future step will be to investigate patterns of vectors for which integer indices cannot be assigned, such as an idealized icosahedron. Along these lines, some classes of quasicrystals have long been classified as Hume–Rothery phases, with the electron count being an important factor.^31^ We are excited about what the MJ electron density for such an array of vectors might predict about the local and long-range atomic arrangements in these materials.

## Supplementary Material



## Notes

Computational details and optimized geometries of the crystal structures; *MJHP*(*E*,2θ) plots for all structures; additional MJ electron density plots for Cu_5_Zn_8_ and Al_4_Ni_3_; dependence of the MJ electron densities for CuZn on the Δ_shell_ and σ parameters (PDF)
