# Supplementary material for: The Mott–Jones Electron Crystal: Patterning Atomic Positions for Pseudogap Formation in Hume–Rothery Phases
Source: Inorg Chem. 2026 Jul 6;65(28):16371–82. doi: 10.1021/acs.inorgchem.6c02050 (PMC13390036; doi:10.1021/acs.inorgchem.6c02050)
Supplement: Supplementary file 1 [file ic6c02050_si_001.pdf]

SUPPORTING INFORMATION

The Mott-Jones Electron Crystal: Patterning Atomic Positions for Pseudogap Formation in Hume-Rothery Phases

Leah C. Garman and Daniel C. Fredrickson*

Department of Chemistry, University of Wisconsin-Madison, 1101 University Avenue, Madison, Wisconsin 53706, United States

*email: danny@chem.wisc.edu

S1. Computational Details and Optimized Geometries for the Body-Centered Cubic Superstructures

Here, the details of the parameters used in the ABINIT DFT calculations are given, along with the optimized atomic coordinates and unit cell parameters. In addition, the definitions of the high-symmetry *k*-points used in the band structure plots are indicated, as well as the scale-factors used in the fat-band representations of the atomic orbital contributions. Isosurface values used for the electron density plots are also provided.

Table S1. Computational details for the geometric optimizations using ABINIT.

Structure	Coarse E Cutoff (Ha)	Fine E Cutoff (Ha)	*k*-point Mesh[a]	No. *k*-points	Coarse FFT Grid	Fine FFT Grid	Total E/Cell (Ha)
CuZn (β-brass)	24	30	12×12×12	84	30×30×30	32×32×32	-425.6926
$AlCu_3$ (BiF_3-type)	22	24	8×8×8	29	40×40×40	40×40×40	-675.5215
$AuAl_2$ (CaF_2-type)	20	26	8×8×8	29	40×40×40	45×45×45	-301.0412
Cu_5Zn_8 (γ-brass)	26	30	6×6×6	16	80×80×80	90×90×90	-5623.2988
Al_4Ni_3 (Ga_4Ni_3-type)	30	30	2×2×2	3	108×108×108	108×108×108	-6713.4590

[a]For all calculations, the *k*-point shift was set to 0.0×0.0×0.0.

Table S2. Computational details for the ABINIT calculations for the MJHP, MJEL, and DOS distribution analysis.

Structure	Coarse E Cutoff (Ha)	Fine E Cutoff (Ha)	*k*-point Mesh	No. *k*-points	Coarse FFT Grid	Fine FFT Grid	Total E/Cell (Ha)
CuZn (β-brass)	24	30	48×48×48	2925	27×27×27	30×30×30	-425.6927
$AlCu_3$ (BiF_3-type)	22	24	32×32×32	969	48×48×48	50×50×50	-2702.0867
$AuAl_2$ (CaF_2-type)	20	26	32×32×32	969	48×48×48	54×54×54	-1204.1661
Cu_5Zn_8 (γ-brass)	26	30	12×12×12	84	80×80×80	90×90×90	-11246.6009
Al_4Ni_3 (Ga_4Ni_3-type)	30	30	6×6×6	20	108×108×108	108×108×108	-13426.9465

Table S3. Optimized cell parameters from ABINIT, converted to the conventional cell.

Structure	*a* (Å)	*b* (Å)	*c* (Å)	*α* (°)	*β* (°)	*γ* (°)
CuZn (β-brass)	2.9618	2.9618	2.9618	90	90	90
$AlCu_3$ (BiF_3-type)	5.8476	5.8476	5.8476	90	90	90
$AuAl_2$ (CaF_2-type)	6.0605	6.0605	6.0605	90	90	90
Cu_5Zn_8 (γ-brass)	8.8684	8.8684	8.8684	90	90	90
Al_4Ni_3 (Ga_4Ni_3-type)	11.4328	11.4328	11.4328	90	90	90

Table S4. Fractional atomic coordinates for CuZn (β-brass).

Wyckoff	Atom	*x*	*y*	*z*
1*a*	Cu1	0	0	0
1*b*	Zn1	0.5	0.5	0.5

Table S5. High-symmetry *k*-points for the path of the orbital-weighted band structure of CuZn (β-brass).

k-point	k_x	k_y	k_z
Γ	0	0	0
X	0	0.5	0
M	0.5	0.5	0
R	0.5	0.5	0.5
Γ	0	0	0

Table S6. Fractional atomic coordinates for $AlCu_3$ (BiF_3-type), converted to the conventional cell.

Wyckoff[a]	Atom	*x*	*y*	*z*
4*a*	Al1	0	0	0
	Al2	0.5	0.5	0
	Al3	0.5	0	0.5
	Al4	0	0.5	0.5
4*b*	Cu1	0.5	0.5	0.5
	Cu2	0	0	0.5
	Cu3	0	0.5	0
	Cu4	0.5	0	0
8*c*	Cu5	0.25	0.25	0.25
	Cu6	0.75	0.75	0.25
	Cu7	0.75	0.25	0.75
	Cu8	0.25	0.75	0.75
	Cu9	0.75	0.75	0.75
	Cu10	0.25	0.25	0.75
	Cu11	0.25	0.75	0.25
	Cu12	0.75	0.25	0.25

[a]Wyckoff labels are provided for one instance of each symmetry-distinct site in this and all following tables of atomic coordinates.

Table S7. High-symmetry *k*-points for the path of the orbital-weighted band structure of $AlCu_3$ (BiF_3-type), defined relative to the primitive cell.

k-point	k_x	k_y	k_z
Γ	0	0	0
X	0.5	0	0.5
L	0.5	0.5	0.5
W	0.5	0.25	0.75
Γ	0	0	0

Table S8. Fractional atomic coordinates for $AuAl_2$ (CaF_2-type), converted to the conventional cell.

Wyckoff	Atom	*x*	*y*	*z*
4*a*	Au1	0	0	0
	Au2	0.5	0.5	0
	Au3	0.5	0	0.5
	Au4	0	0.5	0.5
8*c*	Al1	0.25	0.25	0.25
	Al2	0.75	0.75	0.25
	Al3	0.75	0.25	0.75
	Al4	0.25	0.75	0.75
	Al5	0.75	0.75	0.75
	Al6	0.25	0.25	0.75
	Al7	0.25	0.75	0.25
	Al8	0.75	0.25	0.25

Table S9. High-symmetry *k*-points for the path of the orbital-weighted band structure of $AuAl_2$ (CaF_2-type), defined relative to the primitive cell.

k-point	k_x	k_y	k_z
Γ	0	0	0
X	0.5	0	0.5
L	0.5	0.5	0.5
W	0.5	0.25	0.75
Γ	0	0	0

Table S10. Optimized fractional atomic coordinates for Cu_5Zn_8 (γ-brass), converted to the conventional cell.

Wyckoff	Atom	*x*	*y*	*z*
8*c*	Cu1	0.3278	0.3278	0.3278
	Cu2	0.8278	0.8278	0.8278
	Cu3	0.1722	0.8278	0.1722
	Cu4	0.6722	0.3278	0.6722

	Cu5	0.1722	0.1722	0.8278
	Cu6	0.6722	0.6722	0.3278
	Cu7	0.8278	0.1722	0.1722
	Cu8	0.3278	0.6722	0.6722
12*e*	Cu9	0.3558	0	0
	Cu10	0.8558	0.5	0.5
	Cu11	0	0.3558	0
	Cu12	0.5	0.8558	0.5
	Cu13	0.6442	0	0
	Cu14	0.1442	0.5	0.5
	Cu15	0	0	0.3558
	Cu16	0.5	0.5	0.8558
	Cu17	0	0.6442	0
	Cu18	0.5	0.1442	0.5
	Cu19	0	0	0.6442
	Cu20	0.5	0.5	0.1442
8*c*	Zn1	0.1097	0.1097	0.1097
	Zn2	0.6097	0.6097	0.6097
	Zn3	0.3903	0.6097	0.3903
	Zn4	0.8903	0.1097	0.8903
	Zn5	0.6097	0.3903	0.3903
	Zn6	0.1097	0.8903	0.8903
	Zn7	0.8903	0.8903	0.1097
	Zn8	0.3903	0.3903	0.6097
24*g*	Zn9	0.3137	0.3137	0.0338
	Zn10	0.8137	0.8137	0.5338
	Zn11	0.0338	0.3137	0.3137
	Zn12	0.5338	0.8137	0.8137
	Zn13	0.1863	0.1863	0.5338
	Zn14	0.6863	0.6863	0.0338
	Zn15	0.1863	0.8137	0.4662
	Zn16	0.6863	0.3137	0.9662
	Zn17	0.3137	0.0338	0.3137
	Zn18	0.8137	0.5338	0.8137
	Zn19	0.5338	0.1863	0.1863
	Zn20	0.0338	0.6863	0.6863
	Zn21	0.4662	0.1863	0.8137
	Zn22	0.9662	0.6863	0.3137
	Zn23	0.8137	0.1863	0.4662
	Zn24	0.3137	0.6863	0.9662
	Zn25	0.4662	0.8137	0.1863
	Zn26	0.9662	0.3137	0.6863
	Zn27	0.1863	0.5338	0.1863

Zn28	0.6863	0.0338	0.6863
Zn29	0.8137	0.4662	0.1863
Zn30	0.3137	0.9662	0.6863
Zn31	0.1863	0.4662	0.8137
Zn32	0.6863	0.9662	0.3137

Table S11. High-symmetry *k*-points for the path of the orbital-weighted band structure of Cu_5Zn_8 (γ-brass), defined relative to the primitive cell.

k-point	k_x	k_y	k_z
Γ	0	0	0
H	0.5	-0.5	0.5
P	0.25	0.25	0.25
N	0	0	0.5
Γ	0	0	0

Table S12. Optimized fractional atomic coordinates for Al_4Ni_3 (Ga_4Ni_3-type), converted to the conventional cell.

Wyckoff	Atom	*x*	*y*	*z*
16*a*	Al1	0	0	0
	Al2	0.5	0.5	0.5
	Al3	0	0.5	0
	Al4	0.5	0	0.5
	Al5	0.5	0	0
	Al6	0	0.5	0.5
	Al7	0	0	0.5
	Al8	0.5	0.5	0
	Al9	0.25	0.75	0.25
	Al10	0.75	0.25	0.75
	Al11	0.25	0.25	0.25
	Al12	0.75	0.75	0.75
	Al13	0.25	0.25	0.75
	Al14	0.75	0.75	0.25
	Al15	0.75	0.25	0.25
	Al16	0.25	0.75	0.75
48*f*	Al17	0.0088	0	0.25
	Al18	0.5088	0.5	0.75
	Al19	0.25	0.0088	0
	Al20	0.75	0.5088	0.5
	Al21	0.9912	0.5	0.25
	Al22	0.4912	0	0.75
	Al23	0.25	0.7588	0.5

	Al24	0.75	0.2588	0
	Al25	0	0.25	0.0088
	Al26	0.5	0.75	0.5088
	Al27	0.25	0.9912	0.5
	Al28	0.75	0.4912	0
	Al29	0.5	0.25	0.7588
	Al30	0	0.75	0.2588
	Al31	0.25	0.2412	0
	Al32	0.75	0.7412	0.5
	Al33	0.7588	0.5	0.25
	Al34	0.2588	0	0.75
	Al35	0.5	0.25	0.9912
	Al36	0	0.75	0.4912
	Al37	0	0.25	0.2412
	Al38	0.5	0.75	0.7412
	Al39	0.2412	0	0.25
	Al40	0.7412	0.5	0.75
	Al41	0.4912	0.5	0.25
	Al42	0.9912	0	0.75
	Al43	0.25	0.4912	0.5
	Al44	0.75	0.9912	0
	Al45	0.5088	0	0.25
	Al46	0.0088	0.5	0.75
	Al47	0.25	0.7412	0
	Al48	0.75	0.2412	0.5
	Al49	0.5	0.25	0.4912
	Al50	0	0.75	0.9912
	Al51	0.25	0.5088	0
	Al52	0.75	0.0088	0.5
	Al53	0	0.25	0.7412
	Al54	0.5	0.75	0.2412
	Al55	0.25	0.2588	0.5
	Al56	0.75	0.7588	0
	Al57	0.7412	0	0.25
	Al58	0.2412	0.5	0.75
	Al59	0	0.25	0.5088
	Al60	0.5	0.75	0.0088
	Al61	0.5	0.25	0.2588
	Al62	0	0.75	0.7588
	Al63	0.2588	0.5	0.25
	Al64	0.7588	0	0.75
48*g*	Ni1	0.625	0.1310	0.1190
	Ni2	0.125	0.6310	0.6190

Ni3	0.1190	0.625	0.1310
Ni4	0.6190	0.125	0.6310
Ni5	0.375	0.3690	0.1190
Ni6	0.875	0.8690	0.6190
Ni7	0.375	0.6310	0.3810
Ni8	0.875	0.1310	0.8810
Ni9	0.3810	0.375	0.6310
Ni10	0.8810	0.875	0.1310
Ni11	0.1310	0.1190	0.625
Ni12	0.6310	0.6190	0.125
Ni13	0.1190	0.375	0.3690
Ni14	0.6190	0.875	0.8690
Ni15	0.6310	0.3810	0.375
Ni16	0.1310	0.8810	0.875
Ni17	0.125	0.3690	0.8810
Ni18	0.625	0.8690	0.3810
Ni19	0.8810	0.125	0.3690
Ni20	0.3810	0.625	0.8690
Ni21	0.3690	0.1190	0.375
Ni22	0.8690	0.6190	0.875
Ni23	0.3690	0.8810	0.125
Ni24	0.8690	0.3810	0.625
Ni25	0.875	0.3690	0.3810
Ni26	0.375	0.8690	0.8810
Ni27	0.3810	0.875	0.3690
Ni28	0.8810	0.375	0.8690
Ni29	0.125	0.1310	0.3810
Ni30	0.625	0.6310	0.8810
Ni31	0.125	0.8690	0.1190
Ni32	0.625	0.3690	0.6190
Ni33	0.1190	0.125	0.8690
Ni34	0.6190	0.625	0.3690
Ni35	0.3690	0.3810	0.875
Ni36	0.8690	0.8810	0.375
Ni37	0.3810	0.125	0.1310
Ni38	0.8810	0.625	0.6310
Ni39	0.8690	0.1190	0.125
Ni40	0.3690	0.6190	0.625
Ni41	0.3750	0.1310	0.6190
Ni42	0.8750	0.6310	0.1190
Ni43	0.6190	0.375	0.1310
Ni44	0.1190	0.875	0.6310
Ni45	0.1310	0.3810	0.125

Ni46	0.6310	0.8810	0.625
Ni47	0.1310	0.6190	0.375
Ni48	0.6310	0.1190	0.875

Table S13. High-symmetry *k*-points for the path of the orbital-weighted band structure of Al_4Ni_3 (Ga_4Ni_3-type), defined relative to the primitive cell.

k-point	k_x	k_y	k_z
Γ	0	0	0
H	0.5	-0.5	0.5
N	0	0	0.5
Γ	0	0	0
P	0.25	0.25	0.25
H	0.5	-0.5	0.5

Table S14. Scale factors used to generate the orbital-weighted band structure diagrams.

Structure	Weighting Factor
CuZn (β-brass)	1.6
$AlCu_3$ (BiF_3-type)	1.4
$AuAl_2$ (CaF_2-type)	1.4
Cu_5Zn_8 (γ-brass)	1.0
Al_4Ni_3 (Ga_4Ni_3-type)	0.6

Table S15. Isosurface thresholds for the Mott-Jones Electron Density plots for the {*HH0*} family of vectors.

Structure	Below the E_F ($e^-/Bohr^3$)	Above the E_F ($e^-/Bohr^3$)
CuZn (β-brass)	2.03×10^{-4}	1.80×10^{-4}
$AlCu_3$ (BiF_3-type)	1.64×10^{-4}	1.67×10^{-4}
$AuAl_2$ (CaF_2-type)	2.24×10^{-4}	1.65×10^{-4}
Cu_5Zn_8 (γ-brass)	2.11×10^{-4}	1.80×10^{-4}
Al_4Ni_3 (Ga_4Ni_3-type)	2.64×10^{-4}	1.67×10^{-4}

Table S16. Isosurface thresholds for the Mott-Jones Electron Density plots for the additional {HKL} family of vectors.

Structure	{HKL}	Below the E_F ($e^-/Bohr^3$)	Above the E_F ($e^-/Bohr^3$)
Cu_5Zn_8 (γ-brass)	{411}	1.47×10^{-4}	1.21×10^{-4}
Cu_5Zn_8 (γ-brass)	{$\bar{4}$11}	1.25×10^{-4}	1.45×10^{-4}
Cu_5Zn_8 (γ-brass)	{330}+{411}+{$\bar{4}$11}	4.68×10^{-4}	4.40×10^{-4}
Al_4Ni_3 (Ga_4Ni_3-type)	{215}	4.65×10^{-4}	5.14×10^{-4}

S2. The Mott-Jones Hamilton Population Assessment for the Body-Centered Cubic Superstructures

This section provides the basic MJHP results for the intermetallic structures studied in this work. For each compound, the DOS distribution is lined up with a $MJHP(E, 2\theta)$ plot and $MJHP_{HKL}(E)$ curves for selected families of reciprocal lattice vectors. As described in more detail in Ref. 1, a $MJHP(E, 2\theta)$ plot shows, using a color map, the contributions to the bands at energy E from planewave pairs that interact through different reciprocal lattice vectors (mapped to their diffraction angle, 2θ). The diffraction angle for the Fermi sphere of a hypothetical free electron gas is shown as $2\theta_{FS}$. In an ideal MJ system, the $2\theta_{FS}$ angle would align with a pair of strong features, one stabilizing and one destabilizing, that straddle the Fermi energy (EF) along the energy axis.

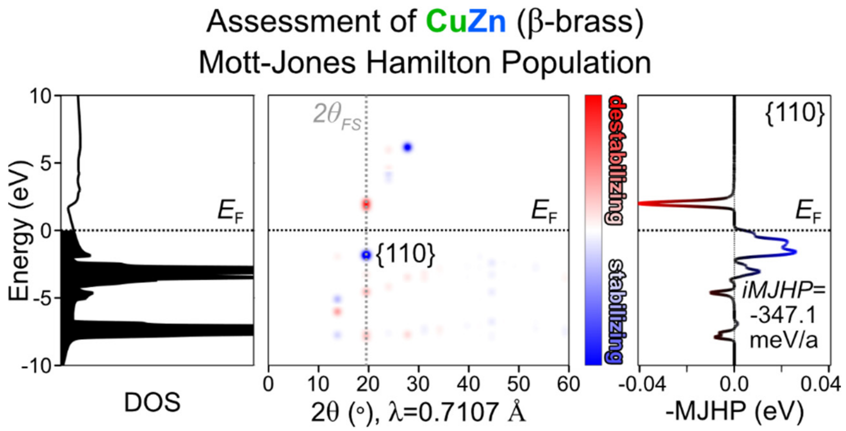

Figure S1. MJHP analysis for CuZn with the electronic DOS distribution, $MJHP(E, 2\theta)$ analysis, and $MJHP_{HKL}(E)$ curve for the {110} family of reciprocal lattice vectors.

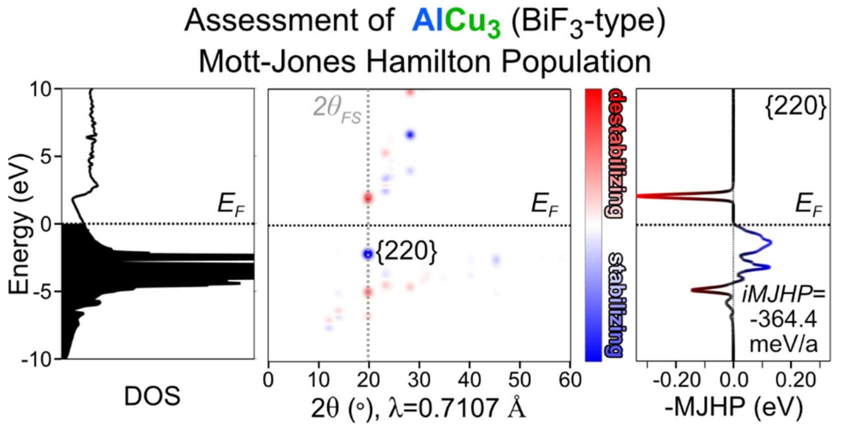

Figure S2. MJHP analysis for BiF_3-type $AlCu_3$, with the electronic DOS distribution, $MJHP(E, 2\theta)$ analysis, and $MJHP_{HKL}(E)$ curve for the {220} family of reciprocal lattice vectors.

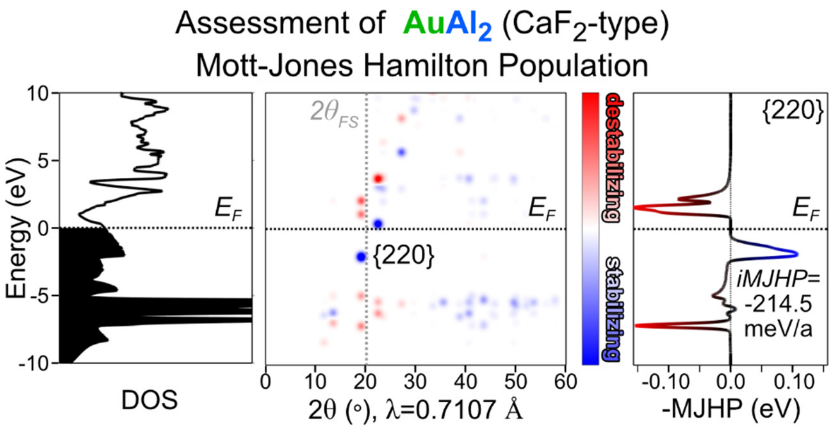

Figure S3. MJHP analysis for CaF_2-type, $AuAl_2$ with the electronic DOS distribution, $MJHP(E, 2\theta)$ analysis, and $MJHP_{HKL}(E)$ curve for the {220} family of reciprocal lattice vectors.

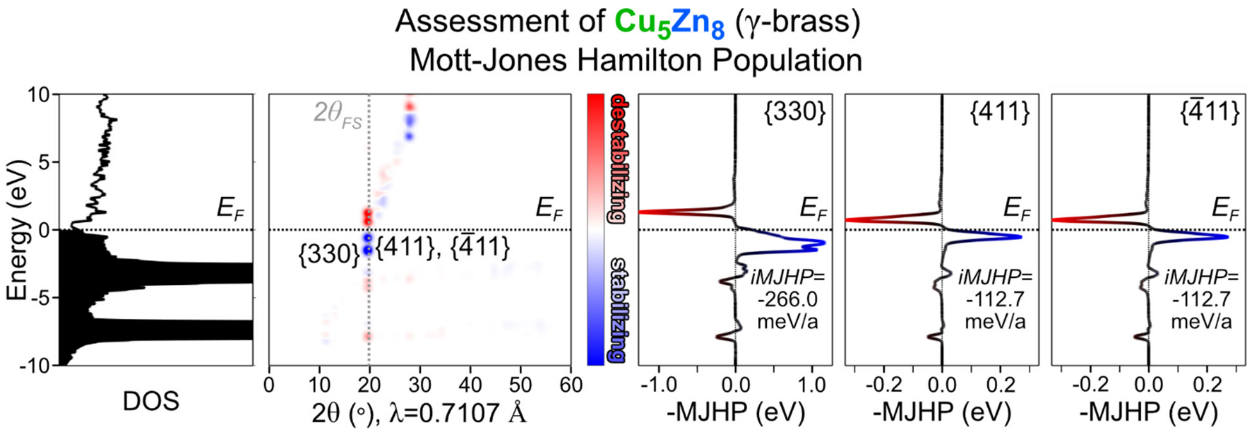

Figure S4. MJHP analysis for Cu_5Zn_8 with the electronic DOS distribution, $MJHP(E, 2\theta)$ analysis, and $MJHP_{HKL}(E)$ curves for the {330}, {411}, and $\{\bar{4}11\}$ families of reciprocal lattice vectors.

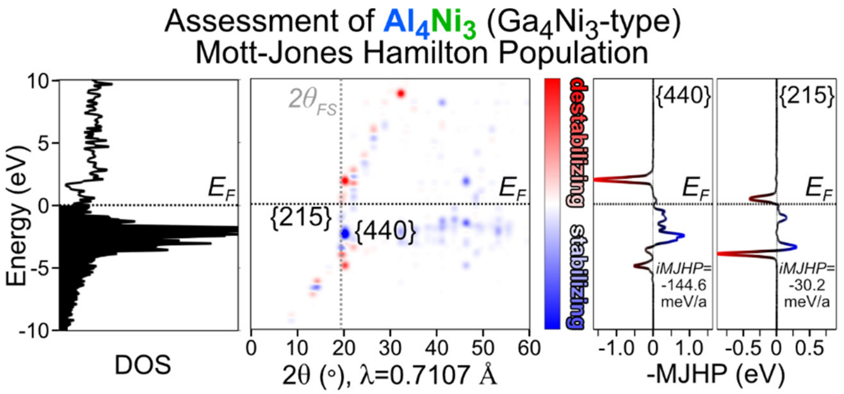

Figure S5. MJHP analysis for Ga_4Ni_3-type, Al_4Ni_3 with the electronic DOS distribution, $MJHP(E, 2\theta)$ analysis, and $MJHP_{HKL}(E)$ curve for the {440} and {215} families of reciprocal lattice vectors.

S3. Additional Mott-Jones Hamilton Population and Mott-Jones Electron Density Plots for Cu_5Zn_8 and Al_4Ni_3

In this section, the Mott-Jones electron densities provided in the main text are supplemented with plots of densities derived from more minor contributors to the MJ stabilization for Cu_5Zn_8 and Al_4Ni_3.

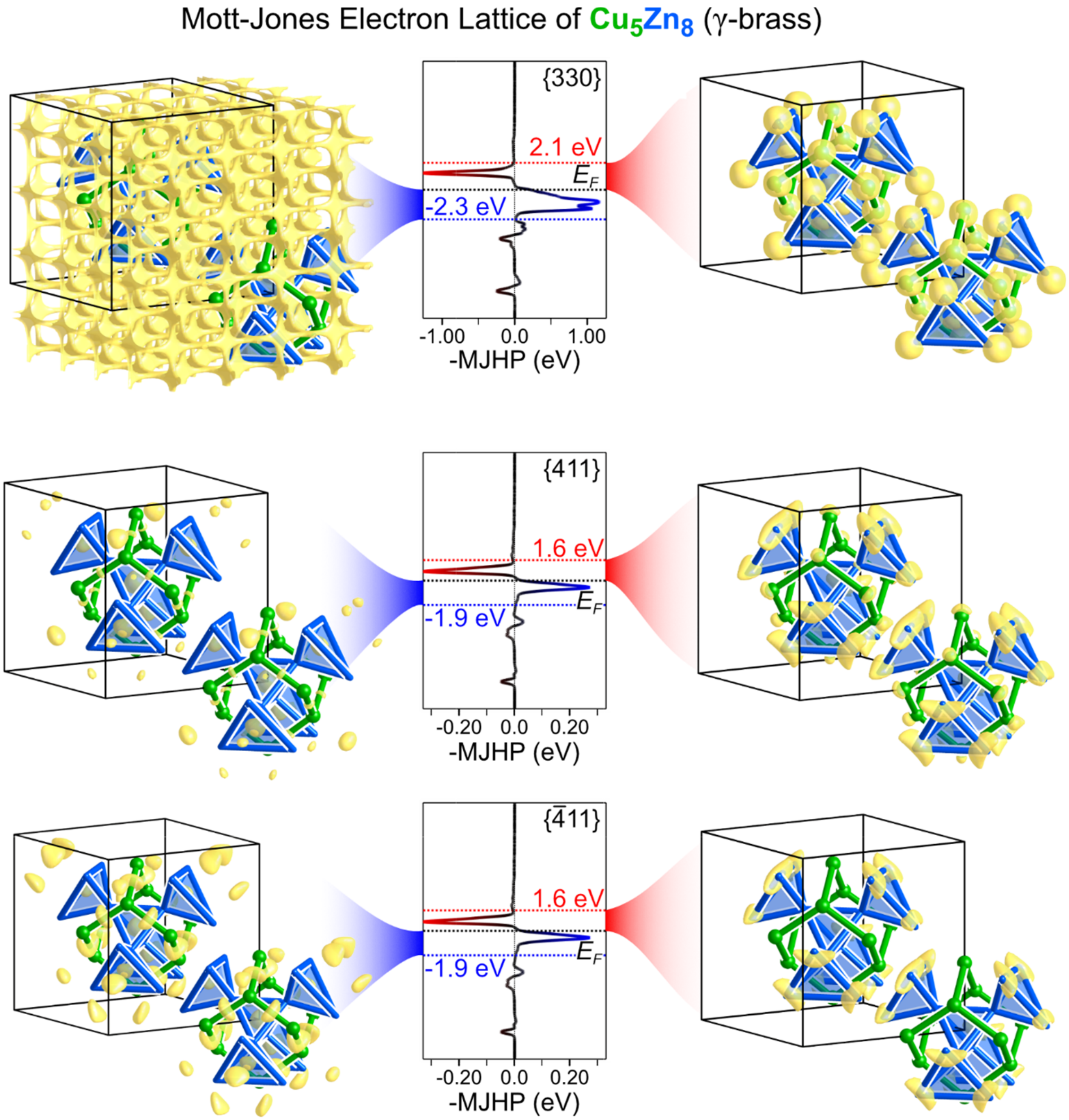

Figure S6. Mott-Jones Electron Lattice for the full, conventional unit cell of Cu_5Zn_8 (γ-brass), derived from the {330}, {411}, and {$\bar{4}$11} families of reciprocal lattice vectors.

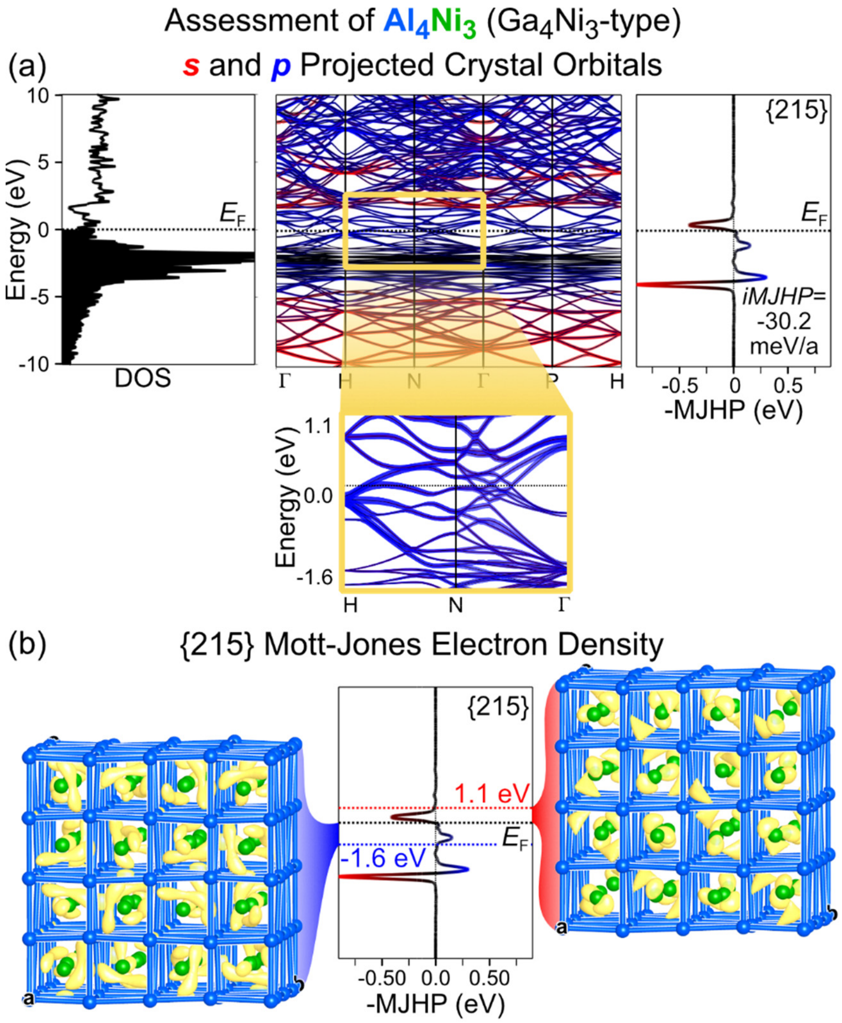

Figure S7. (a) The band structure of Ga_4Ni_3-type Al_4Ni_3, with the *s*- and *p*-type contributions highlighted using a fat-band representation, shown in relation to the DOS distribution and $MJHP_{HKL}(E)$ curve for the minor {215} family of reciprocal lattice vectors, to the left and right, respectively. (b) The MJ electron density for the {215} family of vectors in the stabilizing energy range, -1.6 eV to 0.0 eV (left) and destabilizing energy range 0.0 eV to 1.1 eV (right).

S4. Dependence of the Mott-Jones Electron Lattice Results for CuZn on the Δ_{shell} and σ Parameters.

As described in Section 3.1, the weighting functions used to bring out the planewave pairs involved in the MJ interactions involve two adjustable parameters. Δ_{shell} is the thickness of the spherical shell in reciprocal space within which the two planewaves must reside, while σ controls the steepness of the decay in the weight for a pair planewaves as the magnitudes of the wavevectors differ from each other. In the following, we demonstrate that the qualitative results are largely insensitive to these parameters through plots of the MJ electron densities obtained using different Δ_{shell} or σ values. In addition, tables are provided that give the isosurface levels used in the plots.

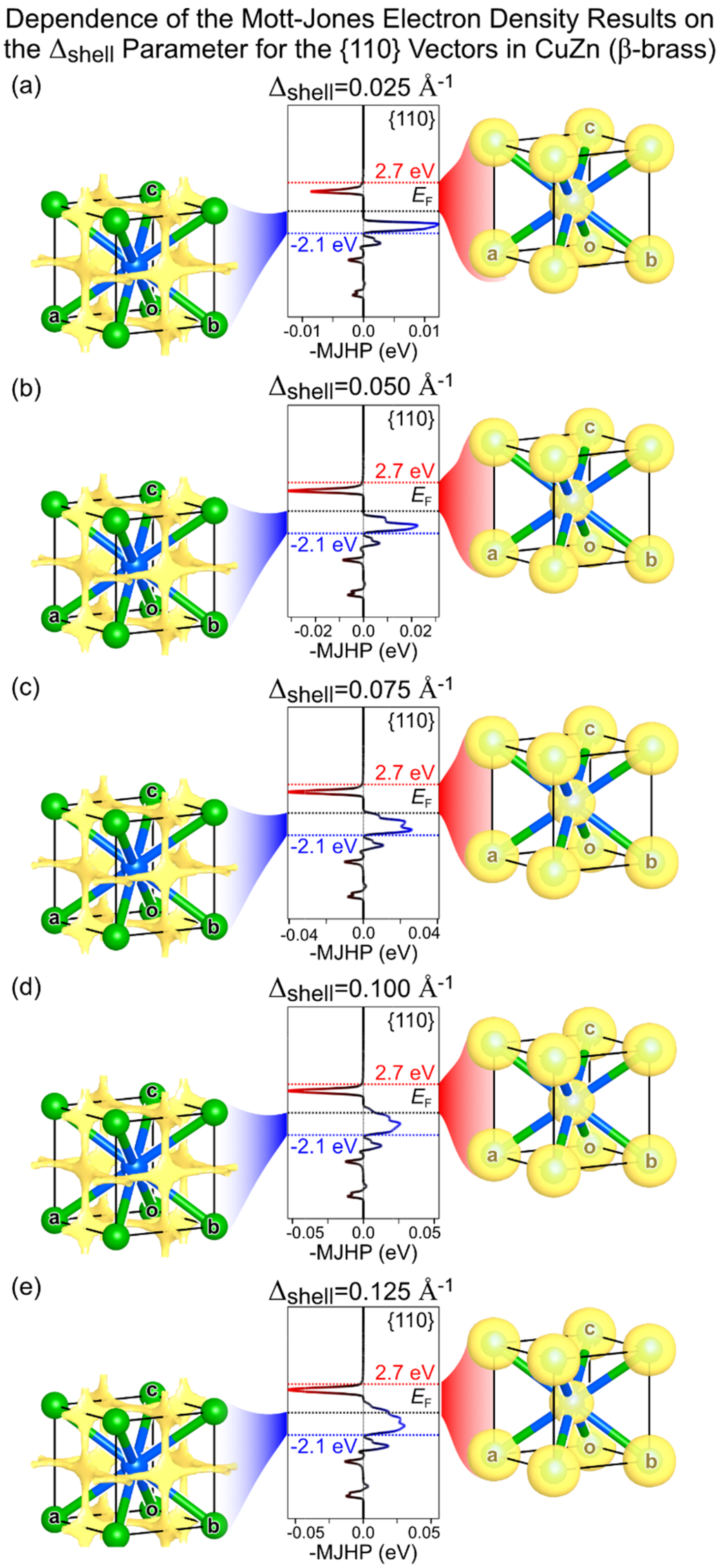

Figure S8. The Mott-Jones electron densities for the {110} family of reciprocal lattice vector for CuZn with varying thickness (Δ_{shell}) parameter values: (a) 0.025 Å^{-1}, (b) 0.050 Å^{-1}, (c) 0.075 Å^{-1}, (d) 0.100 Å^{-1}, and (e) 0.125 Å^{-1}.

Table S17. Isosurface thresholds for the Mott-Jones Electron Density plots for CuZn (β-brass) with varying thickness (Δ_{shell}) parameters.

Thickness (Δ_{shell}) Parameter	Below the E_F (e^-/Bohr^3)	Above the E_F (e^-/Bohr^3)
0.025	2.95×10^{-5}	2.50×10^{-5}
0.050	1.01×10^{-4}	8.80×10^{-5}
0.075	2.03×10^{-4}	1.80×10^{-4}
0.100	3.05×10^{-4}	3.10×10^{-4}
0.125	3.97×10^{-4}	5.00×10^{-4}

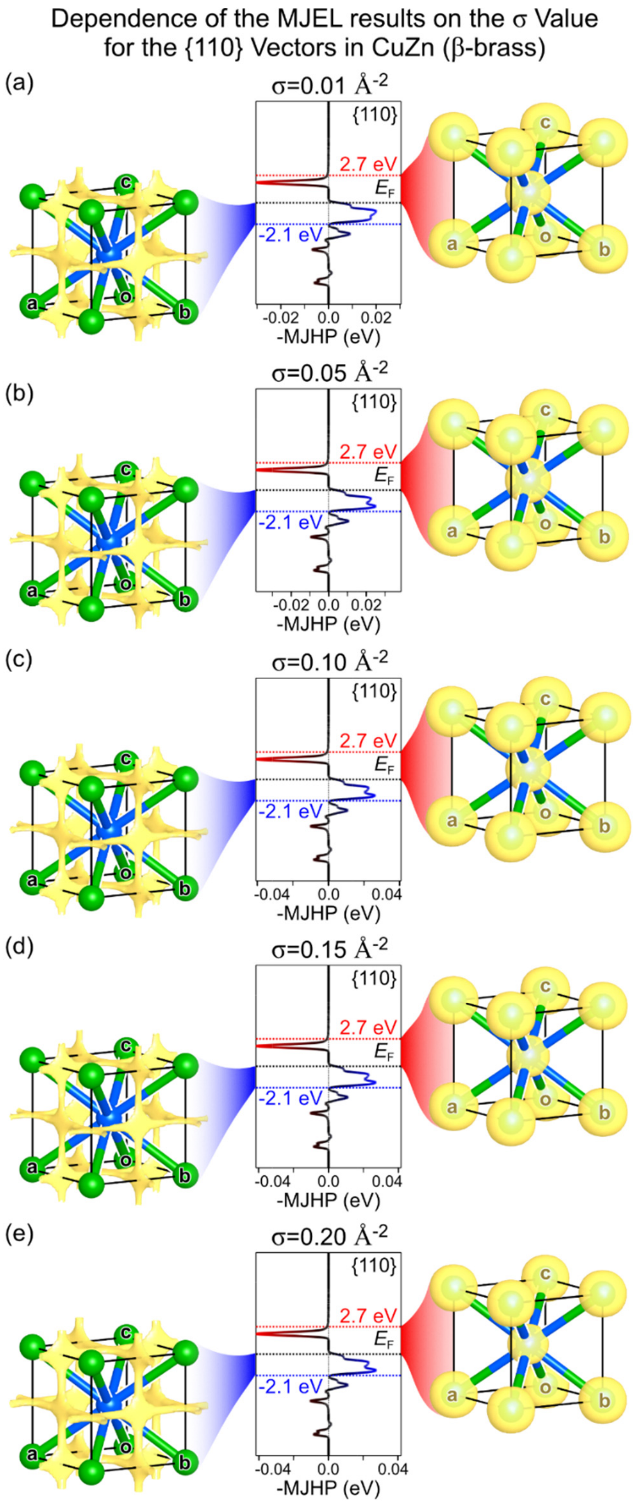

Figure S9. The Mott-Jones electron densities for the {110} family of reciprocal lattice vector for CuZn with varying σ values: (a) 0.01 Å^{-2}, (b) 0.05 Å^{-2}, (c) 0.10 Å^{-2}, (d) 0.15 Å^{-2}, and (e) 0.20 Å^{-2}.

Table S18. Isosurface thresholds for the Mott-Jones Electron Density plots for CuZn (β-brass) with varying σ values.

σ Value	Below the E_F ($e^-/Bohr^3$)	Above the E_F ($e^-/Bohr^3$)
0.025	1.51×10^{-4}	1.30×10^{-4}
0.050	1.95×10^{-4}	1.70×10^{-4}
0.075	2.03×10^{-4}	1.80×10^{-4}
0.100	2.06×10^{-4}	1.85×10^{-4}
0.125	2.07×10^{-4}	1.90×10^{-4}

References

(1) Garman, L. C.; Fredrickson, D. C. The Mott-Jones Hamilton Population: Energetics of Fermi Sphere–Jones Zone Interactions in Intermetallic Structures. *J. Phys. Chem. C* **2024,** *128,* 14442-14457.
